# Prospects of Alkali Metal–Se Batteries and Beyond: From Redox Mechanisms to Electrode Design

**DOI:** 10.1021/acsenergylett.5c00768

**Published:** 2025-04-29

**Authors:** Jimin Park, Hyerim Kim, Arcangelo Celeste, Hyeona Park, Shivam Kansara, Rosaceleste Zumpano, Vanessa Piacentini, Sergio Brutti, Aleksandar Matic, Marco Agostini, Jang-Yeon Hwang

**Affiliations:** † Department of Energy Engineering, Hanyang University, Seoul 04763, Republic of Korea; ‡ Department of Chemistry, “Sapienza” University of Rome, P.le Aldo Moro 5, 00185 Rome, Italy; § Department of Chemistry and Drug Technologies, “Sapienza” University of Rome, P.le Aldo Moro 5, 00185 Rome, Italy; ∥ Department of Physics, Chalmers University of Technology, 41296 Göteborg, Sweden; ⊥ Department of Battery Engineering, Hanyang University, Seoul 04763, Republic of Korea

## Abstract

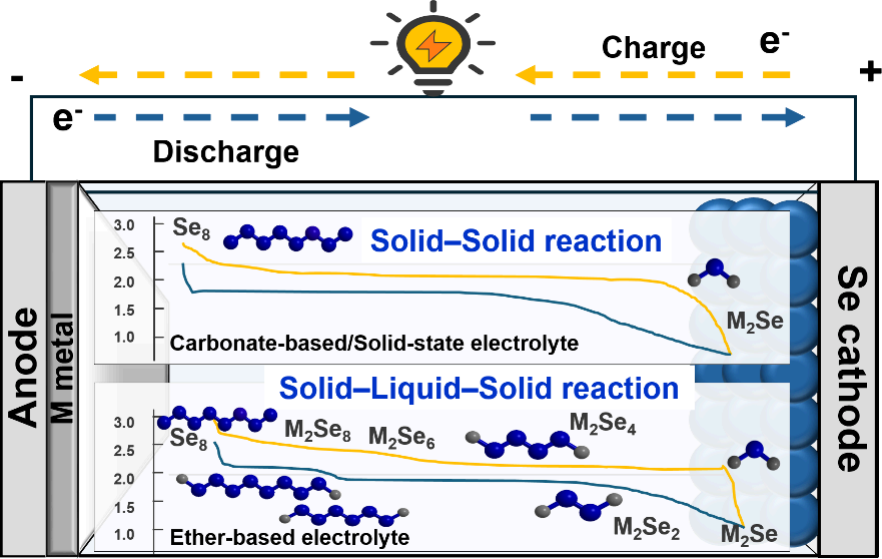

Selenium-based alkali metal systems offer significant potential for surpassing commercial Li-ion systems in volumetric energy density (3,253 vs 1,000 mAh cm^–3^). However, challenges remain in electrode design, solid electrolyte interface stability, and mitigating active material dissolution. This review explores redox mechanisms, electrode architectures, and electrolyte strategies for enhancing performance, with a focus on Li, Na, K anodes and beyond. Advances in computational and experimental studies are discussed, highlighting key issues and future research directions to address scalability and improve stability, making Se-based batteries promising candidates for sustainable energy storage.

Modern energy consumption continues to drive the search for innovative and sustainable energy storage solutions. As traditional fossil fuels face increasing scrutiny owing to environmental concerns and finite reserves, the urgency to adopt clean and efficient alternatives has never been more apparent.^1^ Renewable energy sources hold significant potential for revolutionizing the energy market. However, clean sources like solar and wind are intermittent and require storage systems to redistribute energy on demand.^2^ Li-ion batteries have historically dominated the energy storage market for portable electronics and electric vehicles, owing to their high energy densities. However, the limited availability of Li and related electrode components has prompted the exploration of alternative chemistries. The scarcity and geographical concentration of Li sources, coupled with rising demand, are expected to increase prices and create geopolitical challenges.^3^ Selenium-based alkali metal batteries, incorporating lithium, sodium, and potassium have emerged as promising candidates for next-generation energy storage due to their high theoretical capacity, energy density, and potential for stable, long-term performance. These batteries operate on mechanisms similar to Li-ion technology but benefit from the greater abundance and lower cost of alkali metals.^4^ The multielectron redox reaction of Se, similar to sulfur, offers a higher theoretical capacity than traditional electrodes, with the potential to overcome the limitations of existing battery systems. Additionally, the abundance of Se in the Earth’s crust presents a sustainable and eco-friendly alternative to the rare and costly elements used in conventional cathodes.^5^ While selenium is more abundant than lithium, its extraction and processing present environmental challenges. Sustainable sourcing and recycling strategies are crucial to minimizing the ecological footprint, particularly as Se-based batteries scale up for large energy storage applications.^6^ Selenium offers distinct advantages in alkali metal batteries over sulfur as a cathode material, including higher electrical conductivity, which improves electron transport and enables better rate capability. Moreover, selenium-based batteries demonstrate high volumetric energy density, making them ideal for applications where compact, high-energy storage is essential, such as in portable electronics and grid storage. Fully harnessing the potential of Se electrodes requires a deep understanding of the redox mechanisms in alkali metal batteries and the interfacial processes that occur. Understanding the transformations between different Se species during charge and discharge cycles is essential for optimizing battery performance, capacity retention, and overall stability. Despite these advantages, selenium-based alkali metal batteries face several challenges that limit their commercial viability. Similar to sulfur-based systems,^7−11^ selenium cathodes experience polyselenide formation and dissolution during cycling, leading to the shuttle effect. This phenomenon causes active material loss, low Coulombic efficiency, and rapid capacity fading. Additionally, reactive alkali metals, particularly lithium, are prone to dendrite formation on the anode, which can result in short circuits and pose safety risks. The dendritic growth of alkali metals under prolonged cycling remains a significant obstacle, impacting both anode stability and overall battery performance. In comparison to conventional lithium-ion batteries, selenium-based systems require innovative solutions in terms of electrolyte design, cathode architecture, and interfacial stability to address these issues. Recent research has focused on strategies such as electrolyte modifications to reduce polyselenide dissolution, advanced cathode architectures to confine active material, and protective layers to stabilize the anode. Overcoming these challenges is crucial to fully realizing the potential of selenium-based batteries and establishing them as viable candidates for clean, high-performance energy storage. This review aims to provide a comprehensive outlook on valuable future research directions by offering a deep understanding of the electrochemical mechanisms of Se electrodes in alkali metal batteries, thereby promoting their further development.

## Redox Mechanism

The electrochemical mechanism of Se in alkali metal batteries involves a conversion reaction, where Se is converted to semiliquid polyselenides (PSes) and solid metal selenides, similar to the mechanism observed in sulfur electrodes. The redox mechanism of Se depends on the type of electrolyte and the alkali metal used.

### Lithium

**Figure 1:**
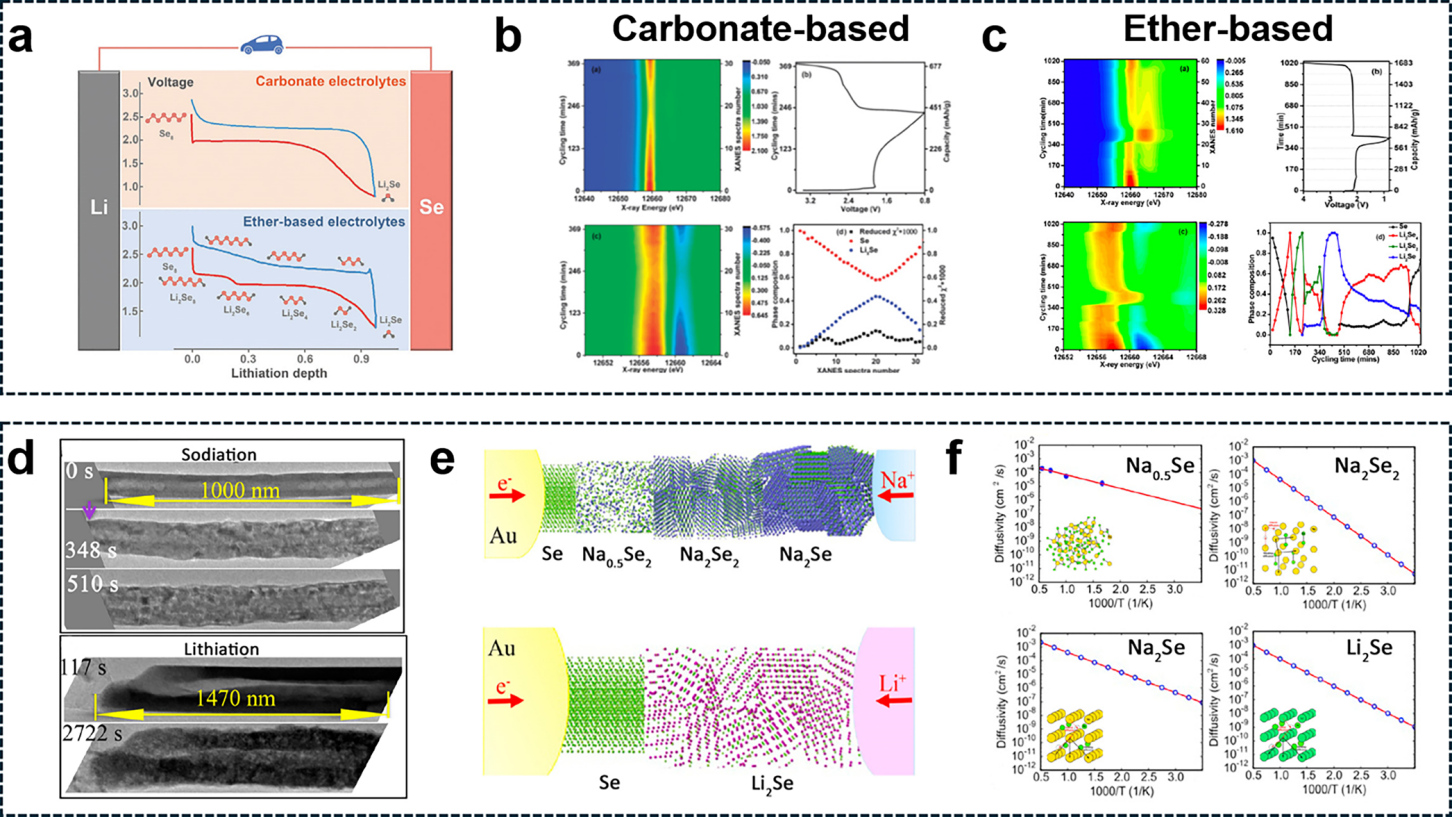
Mechanism of Se in alkali metal batteries. (a) Schematic of Li/Se cell configurations with in-inset voltage profiles using carbonate- (top) and ether- (Bottom) based electrolytes.^12^ (Reproduced with permission from ref (12). Copyright 2021, John Wiley and Sons) (b) *In situ* XANES measurement for a Li–Se cell using carbonate-based electrolytes, showing normalized XANES spectra (top left), voltage profile (top right), derivative of normalized XANES spectra (bottom left), and linear combination fitting of residue values corresponding to phase composition at different states of charge/discharge (bottom right).^14^ (Reproduced with permission from ref (14). Copyright 1996, Royal Society of Chemistry) (c) Normalized XANES spectra of a Li–Se cell in the ether-based electrolyte (top left), voltage profile (top right), derivative of normalized XANES spectra (bottom left), and relative composition evolution of possible phases during cycling.^2^ (Reproduced with permission from ref (2). Copyright 2013, American Chemical Society) (d) Reaction kinetics of Se nanotubes during both sodiation and lithiation processes.^51^ (e) Schematic illustration of sodiation and lithiation in Se nanotubes,^51^ showing the appearance of three different phases during sodiation and one phase during lithiation. (f) Arrhenius plot of the overall diffusion coefficient of Na-ions in Na–Se phases and Li-ions in Li_2_Se phases through a vacancy mechanism.^51^ (Reproduced with permission from ref (51). Copyright 2016, American Chemical Society.)

In Li metal anode–Se cathode systems (LiSeBs) with carbonate-based electrolytes, a solid–solid phase reaction occurs. Conversely, using ether-based electrolytes results in a solid–liquid–solid phase reaction, Figure 1a.^12,13^ Specifically, in carbonate-based solvents, high-ordered species PSes (Li_2_Se_n_, 4 ≤ *n* ≤ 8) are insoluble, leading to the direct reduction of Se to insoluble low-order species (i.e., Li_2_Se_2_/Li_2_Se), as revealed by X-ray absorption near edge structure (XANES) spectroscopy.^14^Figure 1b shows a counterplot of the Se XANES (top left) and the voltage profile of a Se/Li cell using a carbonate-based electrolyte (top right). The derivative of the XANES spectra (bottom left) and the related Se and Li_2_Se phase compositions reveal that the Se edge positions do not shift even at full discharge of the cell (0.8 V), in contrast with Li/Se system in the ether-based electrolyte, Figure 1c.^2^ Further studies on the Li–Se reaction mechanism in carbonate-based electrolytes revealed different pathways, highlighting the solubility of Se and PSes.^15^ To utilize Se in carbonate-based electrolytes, it was confined within porous carbon nanospheres. The nucleophilic Se anion exhibits high reactivity with carbonyl groups, whereas the shells of the nanospheres protect the reaction of Li_2_Se_*x*_ with the carbonyl group by forming a passivated film on the surface during the first discharge.^16^ In addition to lithiation behavior, the crystal structures of Se formed through various steps were investigated. Typically, Se exhibits three types of crystal structures: trigonal (Se_n_, helical polymeric chains), rhombohedral (Se_6_ molecules), and monoclinic phases (Se_8_ rings with α, β, γ configurations).^17−20^ The Se_8_ molecular configuration in chains possesses higher thermodynamic stability than the cyclic Se_8_ configuration.^21^ However, amorphous Se (a-Se) exhibits better electrochemical performance than crystalline Se (c-Se).^22^ The electrochemical performance of a-Se, c-Se, and amorphous/crystalline Se (a/c-Se) cathodes toward Li reveals distinct behaviors.^23^ The a-Se and a/c-Se cathodes exhibit a lithiation process at a two-plateau voltage (∼1.6 and 2.04 V, respectively) through a multistep mechanism. By contrast, the c-Se cathode shows a one-step mechanism with a 1.55 V plateau. The lithiation process in the a-Se process involves Se helical chains that are directly reduced to Li_2_Se owing to their active state and the weakened covalent bonds in the Se_n_ helical chains. Amorphous Se, with its disordered dangling bonds, enables better electrochemical performance and high reactivity with carbonate solvents. However, the strong nucleophilic reaction between chain-like Se and carbonyl groups in carbonate-based electrolytes can lead to capacity fading and short cycle life.^5^ Thus, LiSeBs should be built using amorphous Se cathodes to enhance cell performance. Various strategies to suppress reactivity with carbonyl groups in carbonate electrolytes include designing host materials, nitrogen doping, and incorporating interlayers.^23−25^

In ether-based electrolytes, the charge/discharge behavior of Se follows a multistep reaction, contrasting with the behavior in carbonate-based electrolytes. The electrochemical mechanism typically involves the conversion of Se to high-ordered PSes (Li_2_Se_n_, 4 ≤ *n* ≤ 8), followed by their reduction to low-ordered PSes (Li_2_Se_2_ to Li_2_Se).^1−3,26−37^ These PSes are soluble in ether-based electrolytes, leading to shuttle reactions and significant active material loss.^38^

During discharge, the Se cathode is reduced to Li_2_Se_n_ at approximately 2.1 V, then to Li_2_Se_2_, and finally to Li_2_Se at ∼ 1.9 V. Conversely, during charge, Li_2_Se is oxidized to Li_2_Se_n_ (4 ≤ *n* ≤ 8) at ∼ 2.3 V, then to Li_2_Se_2_, and eventually back to Se. X-ray photoelectron spectroscopy (XPS) has been employed to investigate the formation of PSes through electrochemical reactions, revealing that the ring Se_8_ oxidizes to chain-like Se_8_ after the first charge. In addition, it was investigated the solubility of PSes in ether-based electrolytes using electrochemical impedance spectroscopy (EIS),^39^ finding that these electrolytes exhibit higher resistance than carbonate-based ones. This increased resistance is due to the dissolution of PSes in ether-based electrolytes, which leads to the accumulation of PSes and increased viscosity. To mitigate the negative impact of polyselenide dissolution on electrochemical performance, various strategies have been explored, similar to those developed for carbonate-based electrolytes.^13,17,18,24,25,40−42^ In line with the general ether electrolyte mechanism, Wu et al. reported in 2016 the use of Li_2_Se nanoparticles as a cathode in a conventional ether-based organic electrolyte. The electrochemical behavior of Li_2_Se, akin to that of Li_2_S, displays overpotential during the first charge and shuttling of Li polyselenides. This overpotential can be attributed to the difficulty of extracting Li-ions from crystalline Li_2_Se and the formation of new interfaces. For pure Li_2_Se, a high overpotential of ∼ 3.0 V is required, but Li_2_Se@C composite has a lower overpotential of ∼ 2.4 V at an early stage during the first charge. Several clear differences were distinguished between Li_2_S and Li_2_Se: 1) a lower activation energy barrier for the first charge in Li_2_Se compared to Li_2_S, and 2) a lower overpotential in the first charge for Li_2_Se than for Li_2_S. These results stem from the higher electrical conductivity and ionic mobility of Li in Li_2_Se than those in Li_2_S.^43^

Similar to Li–S batteries, ether-based electrolytes in Li–Se batteries face significant challenges in suppressing the dissolution of polyselenides. The performance of Se batteries depends significantly on the electrolyte used. Recently, DFT calculations have been employed to analyze the lithiation mechanism in amorphous selenium chains, in particular to explain the two discharge plateaus observed in halide-based systems.^44^ Enhancing battery performance requires a deep understanding of the Se battery mechanism in relation to the electrolyte. Indeed, the process of selenium batteries is similar to that of sulfur both in liquid and solid state. Liquid electrolytes facilitate high ionic conductivity and enable efficient ion transport, but they also promote polysulfide and polyselenide dissolution, leading to the well-known shuttle effect. This shuttle effect occurs as soluble polysulfides migrate between the cathode and anode, causing active material loss, reduced Coulombic efficiency, and capacity fading.^12,45^ Moreover, liquid electrolytes tend to permit dendrite growth on the lithium anode, increasing safety risks and further limiting cycle life. Conversely, in solid-state both batteries, the use of solid electrolytes largely mitigates polysulfide dissolution and the associated shuttle effect.^46^ Solid electrolytes, including sulfides, oxides, and polymer-based types, provide a physical barrier that prevents the migration of soluble polysulfides, thus stabilizing the active material at the cathode. Solid-state configurations also inhibit dendrite growth due to the rigidity of the solid electrolyte, which enhances safety and extends cycle life. However, solid-state electrolytes face challenges related to interfacial resistance, which can impede ion transport and lead to higher overall cell resistance compared to liquid-state systems. Interfacial contact between alkali metal electrode and solid-state electrolyte can be optimized by different approaches including: application of surface coatings (e.g., Li_3_PO_4_, Li_3_N, etc.) to enhance ion transfer;^47,48^ incorporation of buffer layers (e.g., amorphous oxide or polymer layers) to mitigate mechanical mismatch;^49^ use of soft solid electrolytes or composite layers to improve physical contact and reduce resistance.^49,50^

### Sodium and Potassium

Few studies have reported on the electrochemical mechanisms of Na/K–Se batteries. For example, the mechanisms in carbonate/ether-based electrolytes have not been thoroughly investigated, and partial mechanistic insights remain scarce (Figure 1d-f).^51−69^ As with lithium ions, selenium has alloying reactions with sodium. It undergoes a transformation during sodiation, first forming amorphous Na_0.5_Se, followed by polycrystalline Na_2_Se_2_ and Na_2_Se. The initial solid-state amorphization, resulting in Na_0.5_Se, is accompanied by a 58% volume expansion. Later, the recrystallization process leading to Na_2_Se_2_ and Na_2_Se induces a significantly larger volume expansion of approximately 336%.^51,70^

The reaction kinetics of Se nanotubes with Na and Li have been studied using real-time transmission electron microscopy (TEM) imaging.^51^ Sodiation of Se nanotubes occurs 4–5 times faster than lithiation, with the solid-state amorphization process being 10 times more rapid than lithiation. This is attributed to the high electronic conductivity and ionic diffusivity of the Na–Se alloy phases formed during sodiation. This electrochemical behavior is consistent with the observed characteristics of K–Se batteries.^63,64,66,69,71,72^ However, the complete reduction of Se cathodes with K is a complicated multielectron reaction depending on the molecular states of Se.^66^

Studies by Yu et al. reported that no intermediate species formed during the first cycle of K–Se batteries. However, they noted the formation of K_2_Se_2_ in subsequent cycles, attributing it to incomplete reactions between Se and K, although they lacked direct evidence to support this behavior.^73^ By contrast, it was proposed that Se directly converts to K_2_Se without forming soluble K–PSes.^74^ Sun and co-workers demonstrate for first K–Se batteries using concentrated ether-based electrolytes can follow distinct reaction pathways, involving the reversible conversion reaction from Se to K_2_Se_*x*_ (x= 5,3,2,1). The highest voltage plateau of K–Se reaction during charge was found at 1.77 V, slightly higher than that observed with conventional carbonate-based electrolytes (∼1.5 V). Furthermore, the average voltage plateau during discharge was found at 1.85 V, a value approaching the theoretical predicted by DFT calculations and about 0.5 V higher than previous reported.^75^

Despite these findings, the underlying mechanisms in both ether- and carbonate-based electrolytes for Na/K–Se batteries remain not fully understood. The differences in ionic radii, binding energies, and polyselenide diffusion among Li^+^, Na^+^, and K^+^ ions significantly affect the behavior of selenium-based batteries. The larger ionic radii of Na^+^ and K^+^ result in greater solubility and mobility of polyselenides, leading to an intensified shuttle effect in Na–Se and K–Se systems.

This increased solubility can improve rate capability by facilitating faster ion transport, but it also presents challenges in retaining active selenium within the cathode, thus impacting long-term cycling stability. Furthermore, energy barriers associated with the reduction of selenium and formation of metal selenides differ across these systems.^3^ Na–Se and K–Se systems generally exhibit lower energy barriers than Li–Se systems, promoting faster reaction kinetics and higher discharge efficiency.^4^ However, the enhanced diffusion coefficients of Na and K polyselenides, while beneficial for charge transport, also heighten the risk of polyselenide dissolution and migration from the cathode, which can lead to capacity degradation.^18^ This comparative analysis underscores why Na–Se and K–Se systems may demonstrate superior redox kinetics and energy density compared to Li–Se, yet they require advanced strategies—such as the use of confinement materials, optimized electrolyte formulations, or interlayers—to control polyselenide dissolution and extend cycle life.^20,45^

### Beyond Alkali-Metals

Beyond alkali metal systems, recent studies have explored Mg, Cu, Al, Zn and Ca–Se batteries as promising candidates.^76^ Mg–Se batteries exhibit multiphase discharge behavior, with a faster transformation of high-order PSes to low-order PSes compared to Mg–S batteries.^77,78^ Cu–Se batteries have reported aqueous Se cathode chemistry, where Cu^2+^ acts as the charge carrier, executing a four-electron reaction sequence (Se → CuSe → Cu_3_Se_2_ → Cu_2-x_Se → Cu_2_Se) during discharge, delivering a high capacity of ∼ 1,350 mAh g^–1^.^79^ Shu et al. explored the redox reaction in Cu–Se batteries from investigating the energy level splitting and binding energies using DFT calculations.^80^ Their findings suggest Cu_2_Se as the most thermodynamically stable phase in the final discharge product, see Figure S1 a,b in Supporting Information (SI) section. Furthermore, the electrochemical behavior of Al–Se batteries, including Se-based six-electron reaction process (Se(−II) ↔Se(0) ↔Se(IV), reveals a superhigh theoretical specific capacity of up to 2,036 mAh g^–1^.^81−85^ However, these studies have some limitations that need to be addressed in future studies. Amine et al. introduced Al–Se electrochemistry based on two-electron transfer process during reduction of Se to Se^2–^, demonstrating performance close to the theoretical capacity of Se. The dissociation and association energies of relevant reactants were investigated using first-principles calculations, with a corresponding energy plots reported in Figure S1c in SI section. DFT calculations indicate that compared to TiO_2_ (101), Se (101) exhibits a higher adsorption energy and a lower diffusion barrier for [AlCl_4_]^−^. TiO_2_@Se-rGO demonstrates excellent electrochemical performance in Al–Se batteries.^86^ Zhi et al. compared the electrochemical performance of Zn–Se batteries using Se/CMK-3 cathodes in both organic and aqueous electrolytes.^87^ Meanwhile, Zhang et al. reported on the redox mechanism of Se electrodes in Ca- based systems. DFT studies reveals a multistep conversion process involving CaSe_4_, Ca_2_Se, and the final product.^88^ This reaction pathway differs from those observed in other Se-metal systems, see Figure S 1d in SI section.

Although the above mechanisms for various cathode-based Se batteries have been suggested, many obstacles and challenges remain in understanding Se batteries, which complicate their commercialization. Extensive research has been conducted using various analyses (such as in situ TEM, Raman spectroscopy, and XRD) to identify the electrochemical mechanism, but no definitive studies have yet been established. In particular, computational analyses, such as DFT calculations, are lacking. Therefore, a more advanced analysis combined with theoretical calculations is required. We believe that studies on Se batteries should incorporate both predictive and supporting experimental results.

### Selenium–Sulfur Hybrid Systems

To combine the advantages of sulfur and Se, selenium sulfide (Se_2_S) as a cathode for Li/Na–Se_*x*_S_*y*_ batteries was reported by Amine’s group in 2012.^1^ The electrochemical reaction mechanism of Li–Se_*x*_S_*y*_ has been extensively investigated.^1,2,5,27,32,89−97^ Similar to Se batteries, Li–Se_*x*_S_*y*_ batteries exhibit general electrochemical reactions depending on the electrolyte solvent (carbonate/ether-based electrolyte). In the carbonate-based electrolyte, Se_*x*_S_*y*_ does not form intermediates, whereas, in ether-based electrolytes, it suffers from shuttle problems owing to the dual soluble intermediate polysulfides and PSes during discharge (Li_2_S_n_ and Li_2_Se_n_, 4 ≤ *n* ≤ 8) Zhang et al. demonstrated a well-organized Li–Se_*x*_S_*y*_ battery system in an ether solvent using operando spectroscopy. This analysis revealed the formation of PSes during cycling and validated the existence and transformation of enhanced polysulfides and PSes (Li_2_S_n_ and Li_2_Se_n_). The formation of Li_2_S_n,_ including Li_2_S_8_, Li_2_S_6,_ and Li_2_S_4_ was confirmed using Raman spectroscopy. Moreover, peaks corresponding to chain-like Se_n_^2–^ (260 cm^–1^) and the active anion free radical Se^2–^ (320–350 cm^–1^) were observed.^98^ Huang synthesized a few-layered molybdenum sulfide selenide (MoSSe) hybrid electrode by sulfur anion doping in molybdenum selenide-based reduced graphene oxide (rGO). This material consisted of a mixture of 1T and 2H phases. During the initial cycle in Na-half cell, a phase transformation reaction led to the formation of 2H–Na_*x*_MoSSe and 1T-Na_*x*_MoSSe, enabling rapid sodium-ion storage.^99^ Yu et al.^64^ developed K-SeS_2_ batteries with long cycle life and high energy density by encapsulating SeS_2_ in a nitrogen-doped self-supporting carbon nanofiber membrane (SeS_2_@NCNFs). After 1,000 cycles at 0.5 A g^–1^, the battery exhibited a high reversible capacity of 417 mAh g^–1^ with 85% retention and nearly 100% Coulombic efficiency.^64^ Cui’s group developed SeS_2_ embedded in ordered mesoporous carbon (named SeS_2_/CMK3) as cathode material, incorporating a foamed copper layer between the cathode and separator to assemble a K-SeS_2_ battery.^100^ Leveraging the synergistic effects of the SeS_2_/CMK3 structure and the Cu interlayer, a novel Mg-SeS_2_ system offering a viable strategy for low-cost, high-area capacity, and high-current rechargeable Mg batteries was proposed. A stainless-steel (SS)-supported lattice-mismatched V–S–Se layered compound (VS_*y*_Se_2-x_-SS) with high Se vacancy concentrations was synthesized by tailoring the S-to-Se molar ratio for ultrafast zinc-ion storage. DFT calculations confirmed that Se vacancies significantly reduce the adsorption energy of Zn^2+^ ions on VS_0.5_Se_2-x_-SS, facilitating a more reversible adsorption/desorption process for Zn^2+^ ions.^101^ Sun et al. successfully addressed the challenge of three-phase interface formation between sulfur, solid electrolyte and the host material (CoMoS_2_@CNT), resulting in the development of high-performance all-solid-state sulfur/SeS_2_ batteries. The battery using SeS_2_ as the active material displayed a feasible areal capacity of approximately 3.7 mAh cm^–2^ at 2.0 mA cm^–2^, retaining 91.8% of its capacity over 400 cycles, highlighting the stable chemistry of the all-solid-state battery featuring CoMoS_2_@CNT.^102^ To suppress shuttle reactions, various Se_*x*_S_*y*_-based cathodes employing separator coatings and hosts with carbon and lithiophilic characteristics have been reported.^89,98,103−106^ Studies using solid electrolytes to prevent the dissolution of PSes in liquid electrolytes have also been reported. PSes are absent during cycling in all-solid-state batteries (ASSBs) that employ solid electrolytes. This suggested a one-step reaction between 2Li + Se → and Li_2_Se. Li et al. first reported an ASSB using Se–Li_3_PS_4_–C as the cathode, Li_3_PS_4_ as the electrolyte, and a Li–Sn alloy anode.^107^ They demonstrated the conversion of Se into Li_2_Se during discharging and the recovery of Se after charging using ex situ X-ray diffraction (XRD) and Raman analyses.

## Computational and Experimental Se-Electrode Design

### Structural Design and Cathode Composition

As discussed in the previous chapter, studies on Se electrodes in alkali metal batteries showed mechanisms and chemistry similar to those of S electrodes, while exhibiting different chemical and physical properties.^31,108^ First, compared to sulfur, Se has a much higher electrical conductivity (10^–4^ vs 10^–15^ ohm^–1^ cm^–1^), which promotes kinetics and electrochemical reactions with alkali metals, reduces the need for conductive additives, and allows for greater active material loading in the cathode. Second, the redox mechanism involving the formation of polysulfide/selenides and the associated “shuttle effect” to the negative electrode surface is less pronounced in Se, resulting in fewer parasitic reactions and reduced active material loss. Finally, the electrochemical potential toward alkali metals is 0.5 V higher for Se than for S, which increases the energy density in electrochemical redox devices.^109^ Despite the “shuttle effect” being less pronounced in Se-based batteries, it remains a notable challenge. For example, the use of pure nanoporous Se as a cathode in Li-ion systems has demonstrated low cell performance owing to pronounced shuttle-side reactions.^110^ Different approaches have been adopted to mitigate the “shuttle effect”^3^ and one of the most exploited is the physical entrapment of Se in porous hosts. Similar to S electrodes,^111^ significant efforts have focused on the synthesis and optimization of conductive porous carbon hosts. The porosity of the host material plays a key role in various functions: (i) enhancing electronic conductivity, (ii) confining an adequate amount of active material at the cathode, (iii) immobilizing high-order selenides to prevent the shuttle effect and loss of the active material, and (iv) accommodating the volume changes of Se during electrochemical reactions with alkali metals. The design of porous carbon structures plays a critical role in optimizing Se electrode performance, but factors such as size, distribution, and morphology are equally important.^112^ Various studies have investigated Se confinement in microspore (*d* < 2 nm)^16,26,53,54,57,63,113−117^ and mesoporous (2 < *d* < 50 nm)^3,75,118−122^ matrices. Materials with micropores efficiently confine Se within the cathode, limiting PSes dissolution. However, they have inherent limitations regarding active material loading. For example, Na–Se batteries utilizing microporous cellulose-derived carbon nanosheets (CCNs) confined with 53% w/w demonstrated a reversible capacity of 613 mAh g^–1^ at 0.1C and 296 mAh g^–1^ at 10C over 500 cycles. In comparison, mesoporous carbon can load a higher content of Se but generally exhibits poorer cycle performance. For example, Se was confined to mesoporous nanocellulose-derived monolithic carbon (Se–NCMC) cathodes in Na–Se batteries, achieving a Se loading of 70 wt %; however, the reversible capacity for Na storage was 511 mAh g^–1^ with 98% retention over 150 cycles.^123^ Hierarchical microand mesoporous carbon hosts have been proposed to confine more active materials to the electrodes.^15,25,56,65,69,71,124−130^ This high-Se electrode was further enhanced by heteroatom doping of the carbon matrix (such as N, O, B, and S).

Using density functional theory (DFT), Islam et al. investigated the binding mechanism of Li_2_Se_n_ on graphene and surface-functionalized Ti_3_C_2_ MXenes. Graphene was used as a reference material to evaluate the binding strengths of Li_2_Se_n_ on functionalized Ti_3_C_2_X_2_ (where X = S, O, F, and Cl). The calculated adsorption strengths of Li_2_Se_n_ on S^–^ and O^–^ terminated Ti_3_C_2_ were found to be higher than those of commonly used ether-based electrolytes, a crucial factor in effectively suppressing Li_2_Se_n_ shuttling.^131^ Furthermore, electronic density distribution studies on the 3DG-CNT@Se material revealed a significant electron density in the interaction between selenium and graphene, suggesting strong chemical bonding.^132^ Wu et al. developed a lignin-derived dual-doped (O and S) hierarchical porous carbon as a selenium host and confirmed, via DFT calculations, the strong affinity of selenides for O and S sites, which help in mitigating the shuttle effect.^133^ As a result, the Se electrode exhibited exceptional cycling stability, retaining a high capacity of 460 mAh g^–1^ after 500 cycles at 0.5 C. Similarly, Li’s group synthesized sulfur- and oxygen-*co*-doped hierarchical porous carbon (SO-HPC) through carbonization and activation. DFT calculations revealed that the binding energy of Li_2_Se to S,O-*co*-doped carbon was significantly higher than that of either S-doped or O-doped carbon alone, indicating enhanced chemisorption of polyselenides and Li_2_Se. Consequently, the assembled Li–Se batteries demonstrated remarkable performance under extreme temperatures, maintaining capacities of 394 and 264 mAh g^–1^ after 400 cycles at 0.5 C at 50 and 0 °C, respectively.^134^

Recently, using DFT calculations it was demonstrated that 2D Ti_3_C_2_T_*x*_ MXene, with its polar interfaces effectively facilitates the chemical immobilization and physical blocking of polyselenides, thereby suppressing the shuttle effect. The unique architecture of Ti_3_C_2_T_*x*_ MXene, integrated atop interlinked nanofibers, ensures continuous electron transfer for redox reactions. As a result, the novel Janus PNCNFs/Se@MXene electrodes exhibit exceptional rate capabilities and long-term cycling stability in both Na–Se and Li–Se batteries.^135^ Additionally, to gain deeper insights into the superior electrochemical performance of Se@NOPC–CNT films as cathodes for Na–Se and K–Se batteries, further studies were conducted.^73^ Furthermore, DFT calculations were performed to model the adsorption behavior of Na_*x*_Se/K_*x*_Se (0 < *x* ≤ 2) on three different substrates: pristine carbon (C), O-doped carbon (OC), and N,O dual-doped carbon (NOC). First-principles calculations at various sodiation stages revealed that the binding energies between Na_2_Se/Na_2_Se_2_ and C, OC, and NOC are −0.41/–0.43, −2.16/–2.90, and −4.08/–4.94 eV, respectively, indicating that OC has a stronger binding affinity than pure C but lower than NOC. A similar trend was observed for K_2_Se/K_2_Se_2_. These findings confirm that N,O dual-doping significantly enhances the chemical affinity between Na_2_Se/Na_2_Se_2_ (and K_2_Se/K_2_Se_2_) and the modified carbon matrix, thereby improving electrochemical performance.^73^ Hu et al. designed a cobalt selenide-sulfide (CoSe_2_–CoS_2_) Mott–Schottky heterostructure catalyst on carbon fibers (CoSe_2_–CoS_2_@CNF). By leveraging the built-in electric field and high catalytic activity of the heterostructure, the CoSe_2_–CoS_2_@CNF effectively reduces the nucleation barrier for polyselenides, enhances the liquid–solid conversion process, and facilitates ordered radial deposition of polyselenides on the carbon fibers. DFT calculations, supported by Gibbs free energy analysis, further demonstrate that the CoSe_2_–CoS_2_ Mott–Schottky heterostructure exhibits the highest catalytic activity, accelerating the rapid conversion of polyselenides.^136^

### Polar Modification of Cathodes

**Figure 2:**
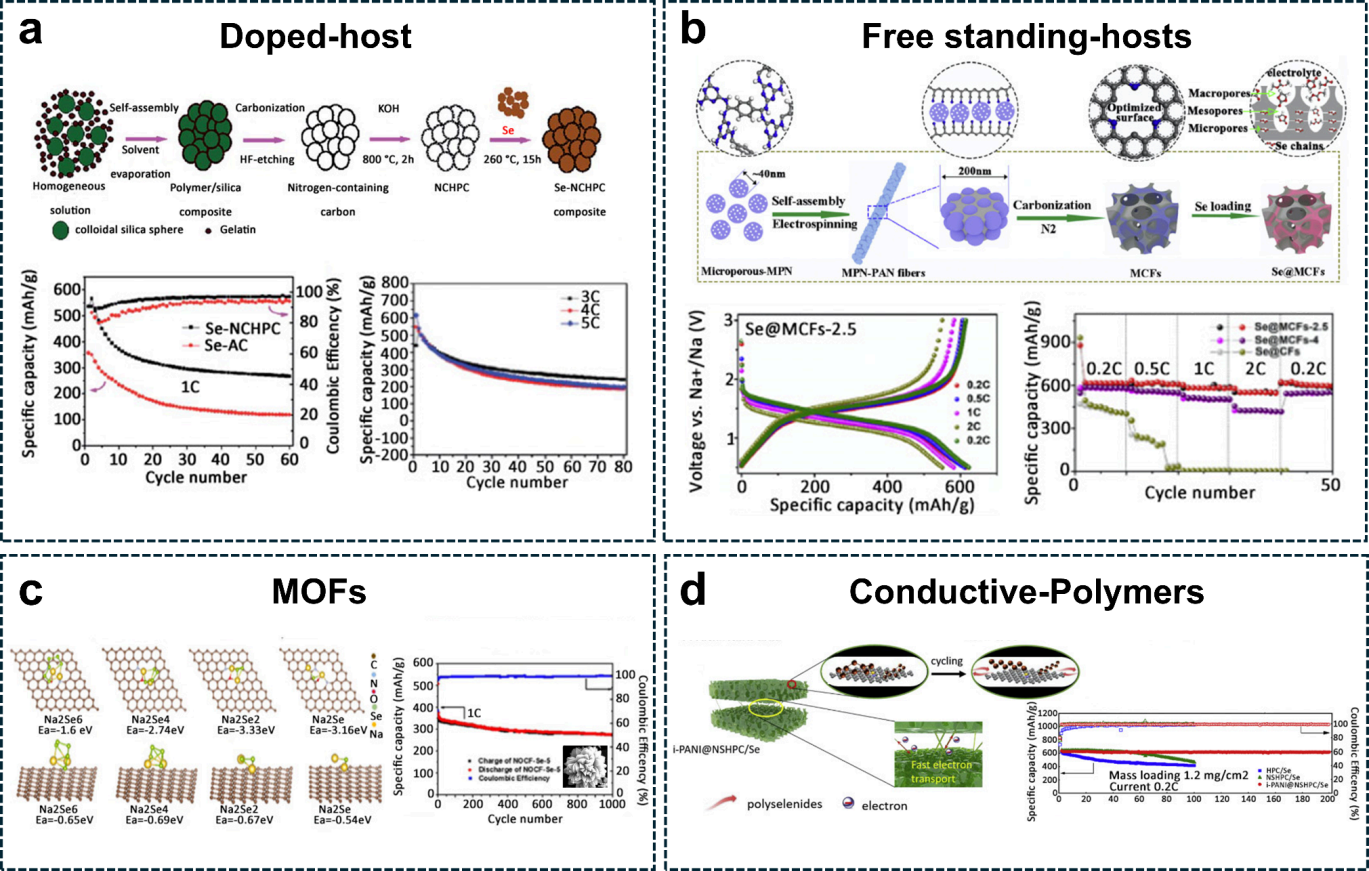
Se electrode design. (a) Schematic illustration of the synthesis procedure (top) for Se-NCHPC, TEM images (bottom left), and the corresponding elemental mapping of C (red), N (orange), and Se (green). Cycling performance (bottom right) of Se-NCHPC and Se-AC in Li/half cells at a current rate of 1C (675 mA g^–1^).^137^ (Reproduced with permission from ref (137). Copyright 2012, Royal Society of Chemistry) (b) Synthesis of free-standing N-doped Murray carbon framework with high Se-loading (top). SEM and STEM images with related EDX mapping of C (yellow), N (red), O (green), and Se (blue). Voltage profiles of the Se@MCF-2.5 cathode and related rate performance (bottom right) in Na/half cells.^126^ (Reproduced with permission from ref (126). Copyright 2021, Elsevier) (c) Adsorption energy of Na-polysulfide calculated through DFT analysis (left). Cycling performance of Se codoped porous flower electrode in Na-based cells.^60^ (Reproduced with permission from ref (60). Copyright 2022, Elsevier) (d) Electrochemical performance of Se-composite with aniline-based polymer in Na-half cells at a current density of 0.2C.^148^ (Reproduced with permission from ref (148). Copyright 2019, Elsevier.)

The presence of heteroatoms improves the binding of polar polyselenide species during the electrochemical process and limits shuttle-side reactions, thus enhancing the battery performance.^137^ Qu et al. reported a novel nitrogen-containing hierarchical porous carbon (NCHPC) to confine Se in Li–Se batteries. Figure 2a shows the synthesis process, which involves the carbonization of a polymer/silica composite, followed by HF etching and mixing with elemental Se.^137^ In the final composite, Se was homogeneously distributed within the carbon host, as confirmed by TEM images and corresponding elemental mapping. From an electrochemical perspective, the authors revealed that the Se–NCHPC composite cathode exhibited a higher reversible capacity (267 mAh g^–1^ after 60 cycles at 1C) compared to a conventional Se/C material. Additionally, the cathode maintained a consistent capacity of approximately 240 mAh g^–1^ across varying current rates, including 3C, 4C, and 5C, showcasing its robust performance under high-rate conditions.

N-containing hollow-core mesoporous-shell carbon spheres (NHCS) demonstrated high anchoring of Se at the cathode side in Li- and Na-based batteries.^138^ A 72 wt % Se-active material was loaded into the NHCS while maintaining excellent cyclability. Freestanding materials have also been proposed by designing hierarchical micro/meso/macroporous compounds composed of N-doped carbon fibers (MCFs). Dong et al. reported the benefits of free-standing electrodes, showing a potential path to reduce inactive material in the Se electrode, reaching a loading of 60 wt % of active material with good capacity retention even at 5C-rate, Figure 2b, ensuring optimal electron and ion transport, as well as Se structural stability.^126^ The synthesis procedure involved the electrospinning of microporous nanoparticles, followed by carbonization, and subsequent Se infiltration in an autoclave. Scanning electron microscopy (SEM) images show the nanofiber morphology, while energy dispersive X-ray spectroscopy (EDX) maps confirm a uniform distribution of all elements. Electrochemically, the Se@MCF-2.5 cathode shows stable voltage profiles as the current density increases from 0.2 to 2C, achieving discharge capacities of 606, 605, 580, and 550 mAhg^–1^ at 0.2, 0.5, 1, and 2C, respectively.

In addition, significant attention has been given to the application of metal–organic frameworks (MOFs) that contain metals. These MOFs are noted for their high surface areas and regular, uniform atom distributions, contributing to their potential as advanced porous structures for battery applications. The favorable characteristics of these frameworks include the ability to tune their composition by selecting appropriate metals and organic ligands.^139^ MOFs can also be doped with abundant heteroatoms, which can enhance their electronic conductivity. Additionally, MOFs possess good hydrophilicity and confinement capabilities,^140^ ensuring fast diffusion of ions and electrons.^52,74,90,141−146^ An N/O codoped flower-like porous host was developed from a Ni-MOF precursor, followed by Se infiltration using two different methodologies: melt diffusion and vapor infiltration.^60^ The vapor infiltration method yielded the best electrode homogeneity by avoiding Se aggregation and achieving an overall loading of 63 wt %. In addition, density functional theory calculations highlighted that the increased polarity of the carbon matrix due to O doping enhances the adsorption of Se and PSes. The performance of the Se electrodes in Li-ion cells showed a capacity of 334 mAh g^–1^ at 0.1C and maintained good reversibility at a 1C rate for over 1,000 cycles, Figure 2c. Transition metal oxides with highly polar surfaces and tunable architectures hold great potential for suppressing intermediate shuttling in Li–Se batteries. To provide guidance for developing metal compound hosts for Li–Se batteries (LSeBs), Choi et al. conducted a computational screening of transition-metal disulfides using DFT calculations. Their study revealed that group IV and V disulfides-specifically VS_2_, NbS_2_, TaS_2_, TiS_2_, ZrS_2_, and HfS_2_-exhibit superior polyselenide entrapment compared to group VI disulfides such as CrS_2_, MoS_2_, and WS_2_. Among them, NbS_2_, TaS_2_, and TiS_2_ emerged as the most promising candidates due to their significantly stronger affinities for polyselenides.^147^ Conductive polymers can also serve as additives to address the PSes shuttle effect and dissolution issues, thereby increasing overall electronic conductivity.^148−152^ In particular, polymers with heteroatoms (e.g., O, S, N) and a highly extended π-delocalized system have been employed to improve Se-electrodes. Zhang et al. reported the designed architecture of an N, S-dual-doped hierarchical porous carbon/Se composite coated with interconnected polyaniline, Figure S2 in SI section and Figure 2d.^148^ The carbon host was synthesized by the carbonization of sodium citrate and thiourea, followed by KOH activation. The N, S-doped C/Se composite was obtained via a simple melt diffusion method. Finally, a polyaniline (PANI) layer with an interconnected structure was grown via the *in situ* polymerization of aniline, resulting in a uniform elemental distribution of C, N, S, and Se, as shown in Figure 2d, which shows the high-resolution transmission electron microscopy-energy dispersive X-ray spectroscopy (HRTEM-EDS) technique. The cycling performance of the composite showed an outstanding capacity retention of 97.3% after 200 cycles, compared to carbon hosts without conductive polymers. The interconnected PANI layer effectively traps PSes within the carbon matrix and acts as a diffusion barrier. Furthermore, the interconnected structure enhances conductivity by linking individual particles, resulting in excellent electrochemical performance. Recent advancements include synthesizing PANI interconnected with the porous carbon matrix of the host through *in situ* polymerization^148^ or on polyacrylonitrile (PAN) fibers through the pyrolysis of PAN/SeS precursors.^153^ Transition metals such (M_*x*_O_*y*_/Se),^150,154−157^ metal selenides (MSe),^78,84,85^ and MXenes (M_n+1_C_n_*T*_m_)^131,135^ have also been investigated for cathodic materials. Introducing metal oxides such as TiO_2_, Al_2_O_3_, NiO, SnO_2_, and ZnO improves the polarity of the Se host and facilitates electrostatic interactions with PSes.^12^ Although oxides offer good adsorption properties for Pses, their low electronic conductivity can limit the rate capability of Se electrodes, necessitating additional conductive additives. A 3D Ni foam foil interlayer was used to improve the electronic properties of the Se/TiO_2_ porous fiber composites, thereby enhancing both electrode cyclability and electronic conductivity.^156^ Another strategy involves the use of a facile deposition technique, namely, atomic layer deposition (ALD), which creates highly uniform thin oxide layers on conductive surfaces. Microporous carbon–Se electrodes were protected using a Al_2_O_3_ thin layer in a Na–Se battery configuration.^157^ The thickness of the metal oxide layer significantly influenced the battery performance, with the optimal result achieved using 10 ALD cycles, promoting the formation of a stable solid electrolyte interface (SEI) layer with fast diffusion of electrons and metal ions. The reversible discharge capacity achieved was 664 mAhg^–1^ at the second cycle, with an 86% retention over 100 cycles. MSes show similar issues related to low conductivity, necessitating the use of additional conductive agents (e.g., porous carbon hosts, carbon nanostructures, and fibers). Despite these challenges, MSe compounds offer significant advantages, such as high specific capacity, low cost, tunability, and catalytic properties, with performance varying based on the type of transition metal used.^158,159^ A notable development in this area is the N-doped porous carbon/ZnSe nanoparticle composite, specifically designed for Zn–Se batteries.^84^ The composite achieved an initial discharge capacity of 172.6 mAh g^–1^ at a current of 300 mAg^–1^ and maintained a capacity of 70.4 mAh g^–1^ after 250 cycles at 500 mAg^–1^ with a discharge voltage of 1.8 V. Although far from the performance in Li or Na-based systems, these results are remarkable compared to other similar works based on Al-storage. The mechanism based on the conversion-alloying reaction was confirmed by ex situ XRD and X-ray photoelectron spectroscopy (XPS) analyses. The results reported using Al anodes are still far from those of other chemistries and need to be widely investigated in the future, both in terms of cell performance and mechanism. To enhance electrical conductivity, surface area, and catalytic properties for promoting PSes adsorption in alkali-Se batteries, researchers have explored MXene-based compounds. This approach leverages a broad range of 2D materials, including transition-metal carbides, nitrides, and carbonitrides.^160^

A notable example involves incorporating Ti_3_C_2_T_*x*_ MXene into Se-infiltrated porous pyrrolic-N-doped carbon nanofibers to create a freestanding cathode for Na-ion batteries.^135^ This design demonstrated a reduction in electron transfer resistance (Rct), which improved charge-transfer kinetics. DFT calculations showed that pyrrolic N significantly enhances PS adsorption. The Na-battery delivered a reversible capacity of 348 mAh g^–1^ at 10C after 5,000 cycles, and the cathode maintained a capacity of 411 mAh g^–1^ even at 20C.

## Electrolytic Configurations for Alkali Se Metal Batteries

### Material Selection for Electrolyte and Formulation

**Figure 3:**
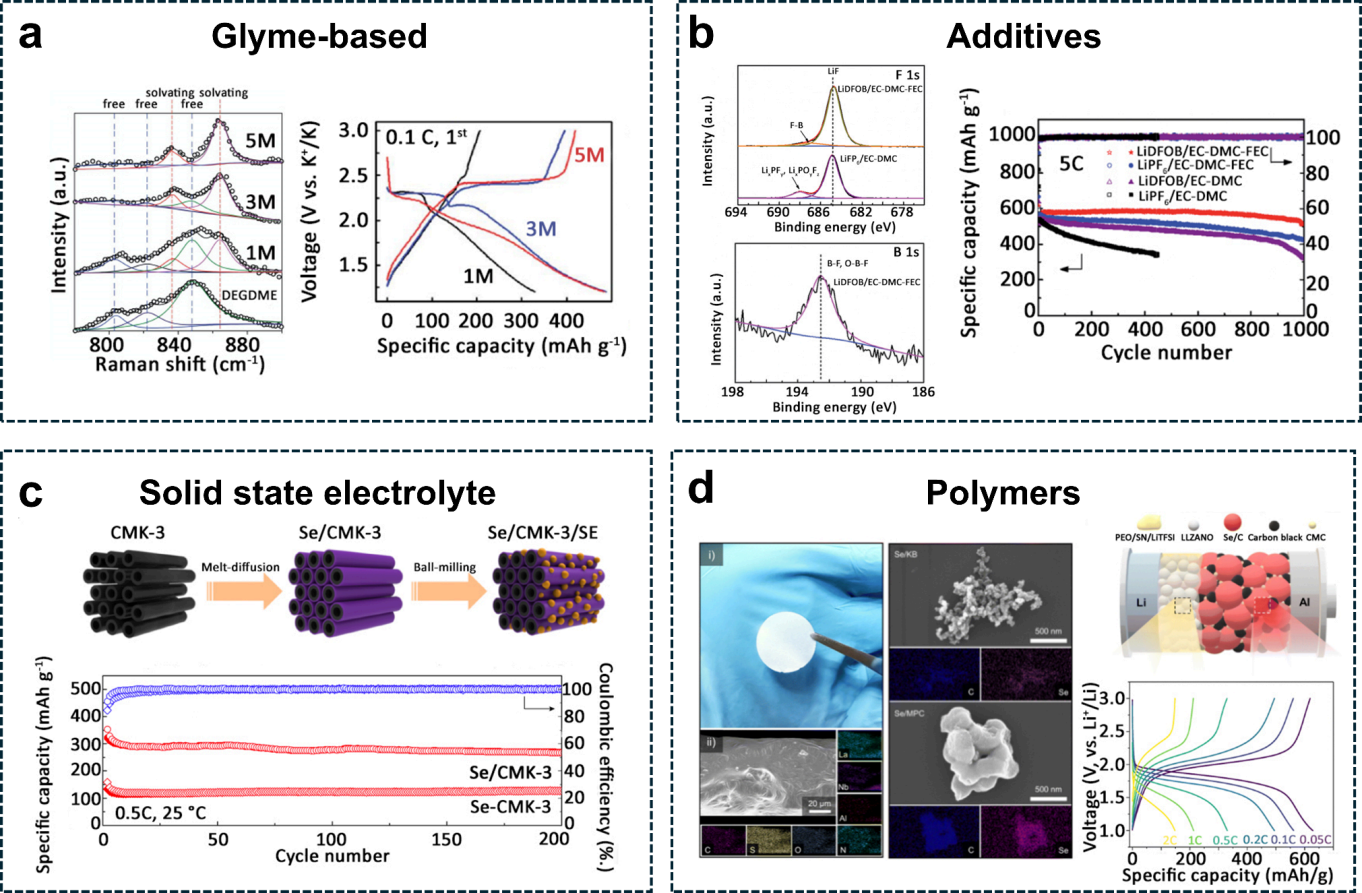
Electrolyte design. (a) Liquid electrolyte: (left) solvation discrepancies revealed by Raman spectra analysis of DEGDME ether-based electrolytes with KTFSI salt concentrations of 1, 3, and 5 M. (Bottom left) Voltage profiles of K–Se cells using KTFSI salt at different concentrations.^75^ (Reproduced with permission from ref (75). Copyright 2010, Royal Society of Chemistry) (b) Additives: Galvanostatic performance of Li–Se cells with different electrolyte configurations including additives at a 5C-rate (1C = 678 mAg^–1^); X-ray photoelectron spectra at the F 1s core level. X-ray photoelectron spectra at the B 1s core level for the cycled Se/MC cathode using LIDFOB/EC-DMC-FEC and LiFP_6_/EC-DMC electrolytes.^115^ (Reproduced with permission from ref (115). Copyright 2012, Royal Society of Chemistry) (c) Solid-state electrolyte: (Top) Synthesis of Se/CMK-3 and mixing with solid-state electrolyte. (Bottom) Cycling performance of Li-battery using Li_10_GeP_2_S_12_ solid-state electrolyte with mixed Se-CMK-3 and annealed Se/CMK-3 at a 0.5C rate and 25 °C.^168^ (Reproduced with permission from ref (168). Copyright 2020, American Chemical Society) (d) Composite polymer: (Top left) visual image of a poly(ethylene oxide) (PEO/LLZANO/LiTFSI/SN-CPE) disk. CPE surface morphology and elemental mapping by EDS. Micromorphologies and EDS elemental mapping results of Se/KB and Se/MPC cathode materials. (Top right) Scheme of cell configuration and (bottom right) rate capability test of the cell using modified polymer electrolyte.^179^ (Reproduced with permission from ref (179). Copyright 2024, Royal Society of Chemistry.)

The electrolyte is a key component of rechargeable batteries, as it significantly affects both the interface at the electrode surface and the overall safety of the device. In Se-alkali metal batteries, low-cost carbonate-based electrolytes can be used with mesoporous carbon-confined elemental Se as the working electrode. However, this combination often results in poor electrochemical performance and rapid capacity fading. To avoid capacity fading, more complex electrodes, such as Se/MWCNT cathodes, are employed in Li-ion cells with an ethylene carbonate/ethylmethyl carbonate (EC/EMC) 1.2 M LiPF_6_ electrolyte mixture.^14^ Capacity fading was investigated through in situ X-ray diffraction (XRD) and X-ray absorption spectroscopy (XPS) analyses, highlighting that the Se crystal structure plays a critical role in the electrochemical performance and capacity fading.^23^ Similar to S-based systems, the use of ether-based electrolytes is also suggested as they can favor the formation of a uniform and compact solid electrolyte interface (SEI), thus preventing the uncontrolled growth of metal dendrites at the anode side.^27,161^ Potassium bis(trifluoromethylsulfonyl)imide (KTFSI)-diethylene glycol dimethyl ether (DEGDME) electrolytes with different salt concentrations (i.e., 1, 3, and 5 M) have been proposed. Raman spectra showed that the intensity of the three vibrational bands associated with DEGDME molecules decreased as the salt concentration increased. Conversely, the Raman signals of solvated DEGDME showed an increasing trend, indicating that at higher salt concentrations (≥3 M), the solvent molecules were coordinating with K^+^ ions, rendering them less available for the PSes shuttle effect.^75^ Although the viscosity of the electrolyte increased with higher salt concentrations, leading to reduced ionic conductivity, K^+^ ions maintained rapid migration even at 5 M KTFSI, Figure 3a. The 5 M electrolyte demonstrated higher specific capacity and better capacity retention compared to the 1 and 3 M electrolytes. Indeed, lower electrolyte concentrations increased the dissolution of redox intermediates, as confirmed by the average Coulombic efficiency (CE) beyond 100% in the 1 and 3 M electrolytes.

Similar results can be achieved by coupling highly salt-concentrated ether-based electrolytes with additives such as fluoroethylene carbonate (FEC)^162^ and/or LiNO_3_,^103^ which react at the anode surface to form an SEI layer that protects the surface against PSes deposition. Similar approaches have also been used in carbonate-based electrolytes to study Li–Se battery performance with different LiDFOB/FEC combinations in EC/DMC.^115^ These results highlight the synergistic effect promoted by the LiDFOB/FEC coupling additives on the capacity and electrochemical stability, which were further analyzed through SEM and XPS characterizations, Figure 3b.^115^ When the cathode was tested at a high current (5C) during the rate capability test, the beneficial effects of both additives were clear. The capacity retention was approximately 88% after 1,000 cycles (511 mAh g^–1^) for LiDFOB/ECDMC-FEC, corresponding to a capacity decay of only 0.012% per cycle. Conversely, electrolytes without additives and those with only FEC or LIDFOB showed the worst performance. The authors attributed these variations in the stability of SEI on the surface of the Li anode and cathode. SEM images showed a compact and smooth Li deposition morphology for LiDFOB/EC-DMC-FEC, compared to the rough and spongy morphology observed with LiPF_6_/EC-DMC. The introduction of FEC promoted greater compactness, while substituting LiPF_6_ with LiDFOB helped prevent the formation of Li dendrites. XPS analysis of the Se/microporous carbon cathode revealed the SEI composition in the LiDFOB/EC-DMC-FEC system, highlighting the presence of LiF, F–B, and O–B–F species. These components are beneficial for protecting both the anode and cathode by suppressing side reactions and enhancing the electrode kinetics Figure 3b.

The risks of explosion and flammability are significant considerations when designing organic solvent-based electrolytes, where both the solvent and salt play crucial roles. Chlorate-based salts, known for their explosive nature, are no longer recommended for use in electrochemical devices.^163^ Valid alternatives include solid-state electrolytes, which fall into two major categories: inorganic and organic. Common inorganic solid electrolytes include perovskites, garnets, Na/Li superionic conductors (NASICON and LISICON, respectively), and sulfide-based electrolytes. These materials are known for their excellent cationic conductivity, high thermal stability, and lack of metal dendrite formation.^164^ They can also prevent shuttle reactions and PSes formation when coupled with Se systems. A Li–Se battery using a solid sulfide electrolyte has advantages in stabilizing the Se electrode performance.^107^ Another promising alternative is related to ionic liquid (IL)-based electrolytes, which are nonflammable, low volatility, and thermally stable electrolytes. Molecular dynamics simulations have investigated Na^+^ transport in various ILs, revealing that cation additives like Li^+^, K^+^, and Ca^2+^ can improve stability Figure S3a in SI section.^165^ Among these, Li^+^ has been identified as the most effective in reducing electrolyte decomposition and enhancing Na-ion transport, offering a viable strategy for stabilizing Na metal anodes Figure S3b-d in SI section.^166^

In summary, for carbonate-based electrolytes, cathodes with stabilized, encapsulated selenium structures are particularly suitable. Carbonate electrolytes are known to react with free selenium or soluble polyselenides, leading to unwanted side reactions and capacity fade. To mitigate this, selenium is often encapsulated within conductive matrices, such as carbon frameworks or polymer coatings, which act as physical barriers to reduce direct contact between the electrolyte and active selenium. For example, selenium encapsulated in porous carbon or mesoporous structures has shown good compatibility with carbonate electrolytes, as these matrices limit polyselenide dissolution and improve structural stability during cycling.^16,18^ Additionally, cathodes with protective coatings, such as metal oxides or conductive polymers, provide a secondary layer that can further reduce reactivity and help maintain stable cycling performance in carbonate systems.^3,45,167^ In contrast, ether-based electrolytes are more compatible with cathodes that employ polyselenide confinement strategies. Ether solvents exhibit greater polyselenide solubility, which supports a higher sulfur loading and enhances redox kinetics. In ether-based systems, the use of carbon hosts and confinement structures, such as metal–organic frameworks (MOFs) or conductive polymers, effectively trap dissolved polyselenides within the cathode, reducing their migration and enhancing cycling stability.

### Interface Engineering in Electrolytes

State-of-the-art solid electrolytes face challenges related to high interfacial resistance and cracking of the electrolyte pellets owing to stress and strain during electrochemical reactions.^168−170^ This resistance impedes ion transport across the solid–solid boundary, reducing overall cell conductivity and rate capability. The rigid structure of solid electrolytes can also limit interfacial contact, especially as electrodes undergo volume changes during cycling, which exacerbates resistance and contributes to capacity fade.^171−173^ Mechanical and chemical stability issues also affect performance. For example, sulfide-based electrolytes are prone to chemical reactions with lithium–metal anodes, forming interphases that increase resistance and reduce ionic conductivity. Furthermore, the mechanical stress from volume expansion and contraction during cycling can lead to cracks and delamination at the interface, affecting overall stability. Oxide-based electrolytes are chemically stable but suffer from limited mechanical flexibility, which may lead to gradual interfacial degradation over repeated cycles.^174^ Another challenge is SEI formation and ion transport. Unlike liquid electrolytes, where stable SEI layers can form naturally, solid-state systems face difficulties in establishing an ideal SEI layer at the interface. While artificial SEI layers have been explored to stabilize these interfaces, creating a conductive, robust SEI across a solid–solid boundary remains challenging.^46^ Inorganic solid-state electrolytes play a crucial role in addressing the challenges of polyselenide migration and overall stability in selenium-based batteries. They act as physical barriers that confine polyselenides within the cathode, preventing their migration to the anode and effectively mitigating the shuttle effect. For example, sulfide-based solid electrolytes create impermeable layers that limit polyselenide dissolution, ensuring better retention of active materials.^175,176^ Additionally, oxide-based inorganic electrolytes are chemically stable, avoiding reactions with polyselenides and forming passivating layers on the anode to suppress cross-contamination and unwanted side reactions. These electrolytes also facilitate efficient lithium-ion transport while blocking polyselenide migration, providing high ionic conductivity and minimizing active material loss.^175,176^ Furthermore, ceramic electrolytes inhibit dendrite growth on the anode through their rigid structures, stabilizing the anode and indirectly reducing the shuttle effect by limiting the reactive surface area. Collectively, these properties highlight the effectiveness of inorganic electrolytes in improving the performance and stability of selenium-based batteries.^177,178^ Hybrid approaches, such as introducing a thin liquid or polymer layer between the solid electrolyte and electrode, have shown promise in reducing interfacial resistance and facilitating ion transport. One addressed these issues by using ordered mesoporous carbon (CMK-3) combined with Se and a solid electrolyte to mitigate volume expansion and reduce interfacial stress, Figure 3c.^168^ Se/CMK-3 composites were mixed with Super P conductive material and a LISICON-based electrolyte, specifically Li_10_GeP_2_S_12,_ through ball milling to promote interface contacts and enhance electron/ion conductivity.

The Li-ion solid-state cell built with this electrolyte showed a reversible capacity of 488.7 mAh g^–1^ after 100 cycles at a 0.05C-rate. Even at a higher current rate of 0.5C, the Se/CMK-3 composite achieved a specific capacity of 269 mAh g^–1^, Figure 3c, with significantly reduced ohmic resistance because of improvements in both electronic and ionic conductivity.

Organic solid electrolytes are solid conductive polymers that suffer from very low ionic conductivities at room temperature, necessitating higher working temperatures to ensure good ion transportation. Using fillers in the polymeric composition (e.g., inorganic nanoparticles, organic plasticizers, and cross-linkers) can enhance the ionic conductivity, but the ionic conductivity still falls below the benchmark value of 10^–3^ S cm^–1^ for practical applications.^193^ Gel polymer electrolytes, formed by adding liquid electrolytes to a solid polymer matrix, can be used in Se-based batteries with good ionic conductivity at room temperature.^180,181^

The coupling of composite polymer electrolytes with Se batteries has been designed to achieve high energy density, low operational temperature, easy manufacturing, and good electrochemical stability, Figure 3d.^179^ A composite polymer electrolyte (CPE) compatible with a Se–C electrode can be prepared using poly(ethylene oxide), Li_6.25_La_3_Zr_2_Al_0.25_Nb_0.25_O_12_ (LLZANO) as a ceramic filler, lithium bis(trifluoromethanesulfonyl)imide (LiTFSI) as a Li salt, and succinonitrile (SN) as a plasticizer. The morphology and microstructure of LLZANO were examined using SEM and energy dispersive X-ray spectroscopy (EDS), respectively. LLZANO is composed of 5–8 μm particles with even distributions from La, Zr, Al, and Nb. The reduction in the crystalline area increased the amorphous content, promoted greater segmented polymer motion, and enhanced ionic conductivity. Thus, the addition of LLZANO improves the ionic conductivity of the solid electrolyte, Figure 3d. The micromorphology of the Se/C composites was identified using SEM images. The corresponding EDS results confirmed the uniform dispersion of Se within the host material. The rate capability test of the Se/C composite in a Li-half cell with a CPE electrolyte showed good performance, with capacities ranging from 640 to 150 mAh g^–1^ at rates between 0.05 and 2C respectively (1C = 675 mA g^–1^) and 25 °C, Figure 3d. Table S1 in SI section, summarizing the advantages and disadvantages of different electrolyte systems has been added to provide a clear and concise overview. The table includes key points for carbonate-based, ether-based, solid-state, and hybrid electrolytes, reporting their benefits and limitations for selenium-based batteries. Among the others, polyselenide dissolution in liquid electrolytes leads to the shuttle effect, causing capacity loss, low efficiency, and degradation due to migration between electrodes and side reactions at the anode. Solid-state and hybrid electrolytes offer solutions but face challenges with ionic conductivity and interfacial stability. Achieving a stable electrolyte-electrode interface is critical, as liquid systems degrade the SEI over time, and solid-state interfaces suffer from mechanical stresses and high resistance. Liquid electrolytes also promote lithium dendrite growth, leading to safety issues, while solid-state systems resist penetration but still struggle with dendrite formation. Limited ionic conductivity in solid-state electrolytes at room temperature hinders rate capability and energy density, requiring advancements in ion transport pathways. High-concentration electrolytes improve SEI stability but suffer from high viscosity, which reduces ionic mobility, and high production costs, limiting their commercial potential.^3,16,18,182,183^

## Negative Electrode

### Anode Materials Modifications and Strategies for Dendrite Suppression

**Figure 4:**
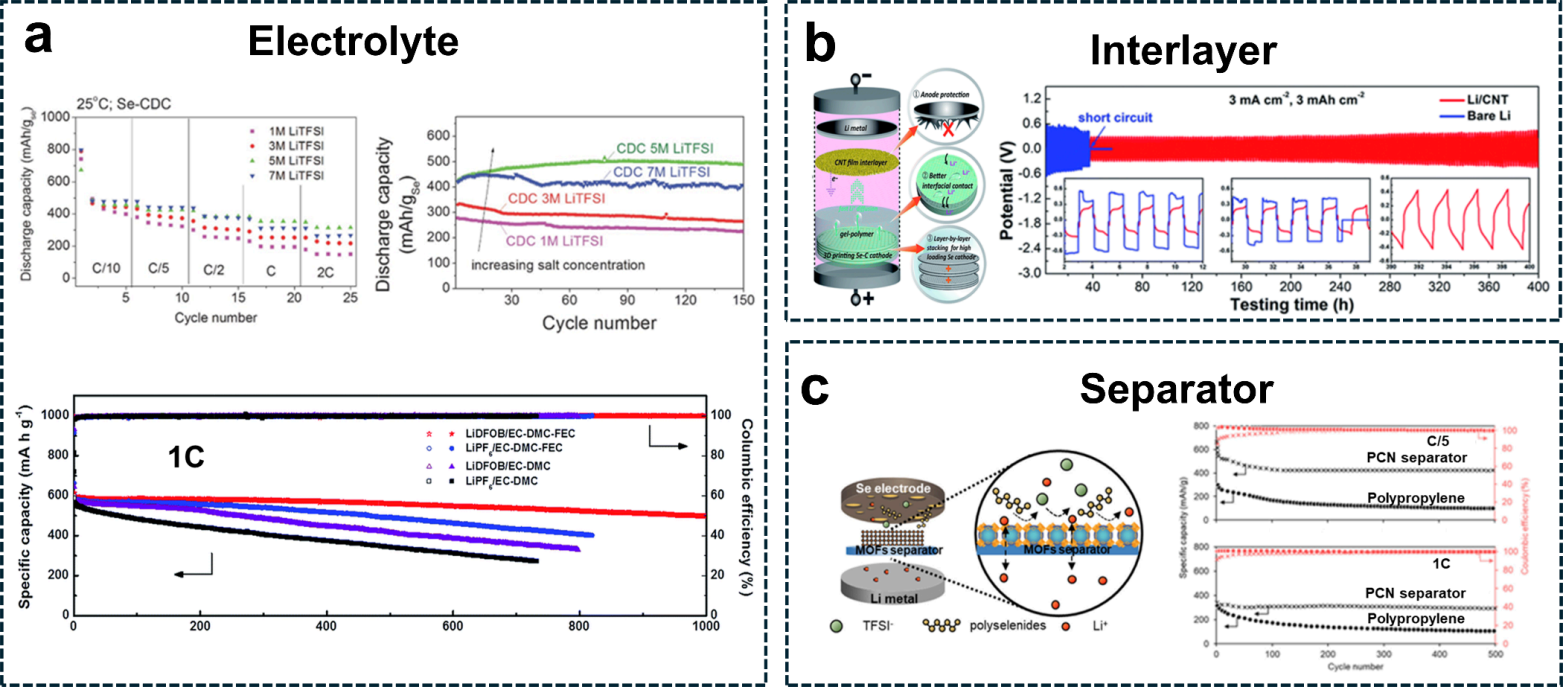
Alkali metal anode design. (a) (Top) Cycling performance with high concentration electrolyte (5 M LiTFSI in DME:DOL (1:1, vol.) with 0.2 M LiNO_3_.^184^ (Reproduced with permission from ref (184). Copyright 2014, John Wiley and Sons.) (Bottom) Cycling performance and Coulombic efficiency of Li–Se cells using different electrolytes at 1C.^115^ (Reproduced with permission from ref (115). Copyright 2012, Royal Society of Chemistry) (b) (Top-left) Galvanostatic discharge/charge voltage profiles of CNT/Li electrodes in symmetric coin cells at a current density of 3 mA cm^–2^ and capacity loading of 3 mAh cm^–2^ for 400 cycles. Electrochemical performance of quasi-solid-state lithium–selenium batteries (QSSLSEBs) with gel polymer electrolytes (GPEs) employing the CNT interlayer at a current density of 1 mA cm^–2^. (Top-right) Schematic illustration of a QSSLSEBs model.^199^ (Reproduced with permission from ref (199). Copyright 2012, Royal Society of Chemistry) (c) Scheme illustrating a cation-selective PCN separator of PCN-250(Fe) metal–organic frameworks coated on a porous polypropylene membrane for suppressing polyselenides shuttle in Li/Se battery systems (Left). Electrochemical performance (rate and cycling) of Li/Se cells with a PCN separator (Right).^42^ (Reproduced with permission from ref (42). Copyright 2021, American Chemical Society.)

Alkali metal–Se batteries generally employ alkali metals (such as Li, Na, and K) as anodes because of their high theoretical capacities compared to nonmetallic anodes.^29,30,184,185^ However, Se metal batteries face significant challenges on the anode side, such as dendritic growth on the metal surfaces during cycling and high electrochemical reactivity. These issues are critical for safety and can cause thermal run-away. In addition, the depletion of electrolytes and side reactions between the metal anode and electrolytes leads to poor safety, low Coulombic efficiency, and capacity fading.^186−188^ Various strategies have been proposed to address these problems, including electrolyte design, solid-state electrolytes, and construction of interlayers and host materials. In 2015, Lee et al. initiated a study on metal anode protection in Li–Se batteries using a high-concentration electrolyte.^184^ They demonstrated that the formation of a LiF-rich solid electrolyte interface (SEI) layer increased the mechanical strength^189^ using an electrolyte composed of 5 M LiTFSI dissolved in DME:DOL(1:1 volume ratio). The SEI layer exhibited higher ionic conductivity, resulting in a capacity retention of ∼ 99.9% of the initial capacity after 150 cycles in the full Li–Se cell, Figure 4a and Figure S4 in SI section.^30,115,184^ LiNO_3_ salt is commonly used as an additive to create a robust SEI layer with high ionic conductivity, suppressing dendrite growth in Li-metal batteries.^190−195^ Many studies on Li–Se batteries have suggested modifying electrolytes with LiNO_3_ additives to improve the electrochemical performance.^29,196^

High-concentration electrolytes have also shown promise in suppressing dendrite formation by promoting the formation of a stable, LiF-rich solid electrolyte interphase (SEI) layer on the anode. In systems with concentrated salts, such as LiTFSI in ether-based solvents, the SEI layer becomes more uniform and dense, which helps stabilize lithium deposition and minimize dendrite growth. This strategy also reduces solvent reactivity with lithium, resulting in improved cycle life and reduced capacity fade. However, increased electrolyte viscosity and higher costs are potential drawbacks of this approach, as discussed in recent studies.^53,162^ Interlayers, including carbon-based structures, lithiophilic coatings, and polymeric films, offer another effective strategy by acting as scaffolds that guide uniform lithium deposition across the anode surface, thereby preventing dendritic growth. Interlayers such as carbon nanotubes (CNTs) and lithiophilic ZnO coatings provide nucleation sites that promote even lithium distribution, reducing localized current density—a primary factor in dendrite formation. Comparative studies indicate that interlayers can achieve more stable cycling performance than high-concentration electrolytes alone. However, the complexity of interlayer integration and the potential increase in internal cell resistance remain challenges.^92,144^

The high theoretical capacity and low electrochemical potential make Li metal the most widely used anode material for high-energy batteries. Consequently, most Li–Se studies employ Li metal as anodes. Integrating Sn-alloys based electrode in Se-systems using solid state electrolyte could prevent dendrite propagation, further enhancing battery safety and longevity.^107^ Thus, a hybrid approach that combines high-concentration electrolytes with interlayers leverages the benefits of both strategies, providing a robust SEI layer through the electrolyte while also guiding lithium deposition via the interlayer scaffold. Such configurations have demonstrated enhanced cycle life and stability, with studies showing that these hybrid systems achieve some of the best results for dendrite suppression and cycling efficiency to date. This comparative analysis emphasizes the effectiveness of different dendrite suppression strategies, providing insights into their respective advantages and limitations. The addition of interlayers, high-concentration electrolytes, or their combination represents a promising pathway to improve cycle life and safety in alkali metal batteries. Liu et al. suggested hybrid electrolytes combining liquid and solid phases to mitigate dendrite growth. This approach leverages the advantages of solid electrolytes^197−199^ and SEI layers formed from liquid electrolytes using a LiNO_3_ additive, Figure 4a.

However, LiNO_3_ has low solubility in carbonate-based electrolytes, limiting its use primarily to ether-based electrolytes.^200^ For carbonate-based electrolytes, highly soluble fluoroethylene carbonate (FEC)^201−203^ solvents are employed as additives to create inorganic LiF-rich SEI layers, Figure 4a. Zhou et al. introduced a novel electrolyte combining FEC solvent with LiDFOB salt to obtain a thin and dense SEI layer.^115^ This FEC-containing electrolyte transformed the fibrous morphology of deposited Li ions into small spherical particles during electrodeposition, resulting in a superior capacity of 511 mAh g^–1^ after 1,000 cycles at a high 5C rate. This FEC-containing electrolyte transformed the fibrous morphology of deposited Li ions into small spherical particles during electrodeposition, resulting in a superior capacity of 511 mAh g^–1^ after 1,000 cycles at a high 5C rate.

### Artificial SEI Layer Formation

In addition to liquid electrolytes, Guo’s group introduced in situ gelation in Li–Se batteries for the first time, utilizing the cationic ring-opening polymerization principle.^204^ Polymerization, generated from an electrochemical reaction between the 1,3-Dioxolane (DOL) solvent and the LiPF_6_ salt, transformed the liquid electrolytes into gel polymers during cycling. These gelation electrolytes, deposited on the Li metal surface, restrained the side reactions between the Li metal and the electrolytes, improving capacity retention to 80%, compared to 60% retention in liquid electrolytes.

Li–Se batteries have been developed using various electrolytes, such as polymers, sulfides, oxide solid electrolytes, and liquid electrolytes.^168,205,206^ Lee’s group presented a “one cathode design” cell, combining a cathode with polymer electrolytes electrodeposited on it to simultaneously control the shuttle effect and dendrite formation.^205^ Despite the promising properties of these materials, challenges remain, particularly in terms of their poor mechanical strength and inability to effectively suppress Li dendrite growth at high Se loadings and current densities.^207,208^ Recently, Gao et al. reported that a 3D-printed interlayer could inhibit dendrite growth, Figure 4b.^199^ Symmetric cell tests demonstrated the effectiveness of the constructed interlayer in suppressing dendritic growth.^199^ Sathitsuksanoh et al. proposed a separator coated with MOF to fix Lewis base sites, enabling reversible plating/stripping behavior, Figure 4c. This approach blocks the TFSI^–^ anion, a Lewis base from reaching the anode side selectively interacting with the separator surface during cycling. This mechanism promotes stable and uniform Li electrodeposition on the Li metal anode, achieving a high Li^+^ transference number.^42^ Na-metal anodes are also being explored due to the abundance and high specific capacity (∼1,166 mA h g^–1^) of Na. However, challenges such as surface instability, dendritic growth, and volumetric changes have hindered the commercialization of Na–Se batteries.^209^ Hu et al. suggested a dendrite-free anode with strong sodiophilic properties, derived from an N,O-codoped carbon host using a Zn-based MOF (denoted NOC/Na). The NOC/Na host exhibited higher ionic conductivity, providing more Na^+^ deposition sites for uniform plating and reducing the effective current density, which in turn stabilized the Na metal during cycling.^210^ The NOC/Na structure mitigated dendritic growth during cycling, and the full cell incorporating a Ni-SA/NOC cathode, retained a capacity of 213 mAh g^–1^ (1026 mAh cm^–3^) even after 1,000 cycles at 1C rate.

Song et al. demonstrated that the copresence of Py_1,4_TFSI and LiNO_3_ in the electrolyte can protect the lithium anode by forming a stable solid electrolyte interphase (SEI) rich in LiF, LiN_*x*_O_*y*_, and conductive Li_3_N. This smooth SEI minimizes morphological changes to the lithium anode during cycling, effectively mitigating lithium dendrite growth and undesirable side reactions with soluble cathode intermediates.^211^ The concept of bilayer artificial SEI (BL-SEI) introduced by Zhang’s group highlights the potential of combining graphitic layers (GLs) with inorganic layers (ILs) such as LiF and Li_2_CO_3_ to enhance lithium metal anode stability. The GLs provide high mechanical strength, mitigating stress from nonuniform lithium deposition, while the ILs prevent corrosion from the electrolyte. DFT-based charge density difference analysis, Figure S5a-d SI section, and stress–strain evaluations, Figure S5e-h SI section, further reveal the stability, ionic conductivity, and mechanical resilience of the graphene/LiF ⟨111⟩ BL-SEI, positioning it as promising artificial SEI candidate. Expanding on this theoretical framework, future studies could explore the design of artificial SEIs for selenium batteries.^212^

Alkali metals are preferred as anodes in Se batteries owing to their high theoretical capacities compared to other anode materials, such as graphite and hard carbon. Nonmetallic anodes require an additional lithiation step and still exhibit lower energy densities than alkali metal anodes.^213,214^ For example, Si anodes in Se batteries undergoes significant volume changes—up to 400%—during lithiation and delithiation, which can hinder the capacity and long-term cycling stability of Se batteries.^185^ While alkali metals offer advantages in terms of energy density, their use as anodes in Se batteries poses challenges, including electrolyte depletion, volume expansion, and dendrite formation during charge/discharge cycles.

In summary, this review explores the key advancements and persistent challenges in the development of Se alkali metal batteries, including redox mechanisms, electrode designs, and electrolytic configurations. Innovative cathode materials such as conductive porous carbon hosts, MoFs, and conductive polymers have shown improved performance and stability by mitigating issues such as polyselenide dissolution and shuttle effects. On the anode side, despite the high theoretical capacities of alkali metals (Li, Na, and K), challenges such as dendrite formation, electrolyte depletion, and volume changes remain. Strategies such as high-concentration electrolytes, solid-state electrolytes, and protective interlayers have demonstrated the potential for enhancing anode stability.

Table S2 in SI section reports visual understanding of the advantages and limitations of each alkali metal–selenium system discussed and highlights the specific challenges each one faces. The included table presents these performance differences across various parameters, such as energy density, cycle stability, and reaction kinetics. This comparative approach underscores why, despite their inherent advantages, each alkali metal–selenium system encounters unique challenges that must be addressed in the future to enhance their practical applications. Future research should focus on optimizing the interactions between the electrodes and electrolytes, further developing hybrid electrolytes that combine liquid and solid-state benefits to enhance ionic conductivity and safety, addressing SEI formation and growth, further exploring Na and K as viable and cost-effective anodes. Advanced electrode design by developing 3D-structured or porous materials to improve electronic conductivity and accommodate volume changes for better cycling stability should be explored. Integrating individual strategies for dendrite suppression, including high concentration electrolytes and interlayer design should address challenges on practical applications, i.e. high loading and long cycling. Sustainability exploring the recycling and reuse of waste materials should be improved. These approaches could lead to more sustainable and commercially viable Se-based batteries. In conclusion, although significant progress has been made, continued innovation in material engineering and electrolyte formulation is crucial to fully leverage the potential of Se-alkali metal batteries. Addressing the remaining challenges could make these batteries key technologies for high-performance and sustainable energy storage.

## Supporting Information Available

DFT calculation; binding energy; energy plot; voltage curves; schematic of manufacturing Se-composite; HR-TEM/EDX; LUMO energy level; cycle performance; electrode potential; charge density; comparison of advantages and disadvantages of different electrolyte systems for Se batteries; comparison of alkaline Se systems in terms of theoretical capacity, cycle-life, polyselenide solubility, diffusion coefficient and redox kinetics (PDF)
