# Supplementary material for: Prospects of Alkali Metal–Se Batteries and Beyond: From Redox Mechanisms to Electrode Design
Source: ACS Energy Lett. 2025 Apr 29;10(5):2512–31. doi: 10.1021/acsenergylett.5c00768 (PMC12070738; doi:10.1021/acsenergylett.5c00768)
Supplement: Supplementary file 1 — nz5c00768_si_001.pdf [file nz5c00768_si_001.pdf]

Supporting Information

Prospects of Alkali Metal-Se Batteries and Beyond: from Redox Mechanisms to Electrode Design

Jimin Park,[a,#] Hyerim Kim[a,#] Arcangelo Celeste[b,#], Hyeona Park[a], Shivam Kansara[a], Rosaceleste Zumpano[c], Vanessa Piacentini[b], Sergio Brutti[b], Aleksandar Matic[d], Marco Agostini*[,c] and Jang-Yeon Hwang*[,a,e]

[a]Department of Energy Engineering, Hanyang University, Seoul 04763, Republic of Korea.

[b]Department of Chemistry, "Sapienza" University of Rome, P.le Aldo Moro 5, 00185 Rome, Italy.

[c]Department of Chemistry and Technology of Drugs, "Sapienza" University of Rome, P.le Aldo Moro 5, 00185 Rome, Italy.

[d]Department of Physics, Chalmers University of Technology, 41296 Göteborg, Sweden.

[e]Department of Battery Engineering, Hanyang University, Seoul 04763, Republic of Korea.

[#]These authors contributed equally to this work.

*Corresponding authors: marco.agostini@uniroma1.it; jangyeonhw@hanyang.ac.kr

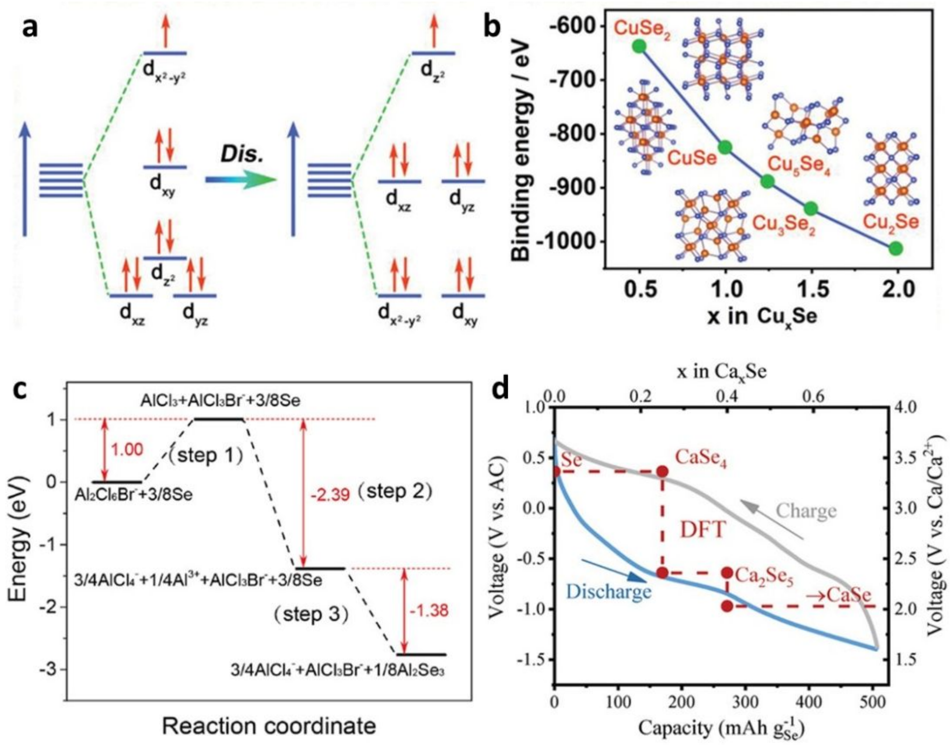

Figure S1| **a** 3d orbital diagrams in $[Cu(H_2O)_4]^{2+}$ (left) and CuSe (right). **b** The binding energy and schematic molecular structure of Cu_xSe.[1] Reproduced with permission from ref. 1. Copyright 2022, John Wiley and Sons. **c** Energy plots of dissociation and association of reactants for the operation of Al-Se battery[2] (Reproduced with permission from ref. 2. Copyright 2019, Elsevier) and **d** computed equilibrium voltage curves and experimental curves comparison of Se electrode in Calcium battery system.[3] Reproduced with permission from ref. 3. Copyright 2022, John Wiley and Sons.

(1) Zhang, J.; Zhang, X.; Xu, C.; Yan, H.; Liu, Y.; Xu, J.; Yu, H.; Zhang, L.; Shu, J. Four-Electron Transfer Reaction Endows High Capacity for Aqueous Cu–Se Battery. *Adv. Energy Mater.* **2022**, *12* (19), https://doi.org/10.1002/aenm.202103998.

(2) Liu, S.; Zhang, X.; He, S.; Tang, Y.; Wang, j.; Wang, B.; Zhao, S.; Su, H.; Ren, Y.; Zhang, L.; Huang, J.; Yu, H.; Amine K. An Advanced High Energy-Efficiency Rechargeable Aluminum-Selenium Battery. *Nano Energy* **2019**, *66*, 104159, https://doi.org/10.1016/j.nanoen.2019.104159.

(3) Zhou, R.; Hou, Z.; Liu, Q.; Du, X.; Huang, J.; Zhang, B. Unlocking the Reversible Selenium Electrode for Non-Aqueous and Aqueous Calcium-Ion Batteries. *Adv. Funct. Mater.* **2022**, *32* (26), https://doi.org/10.1002/adfm.202200929.

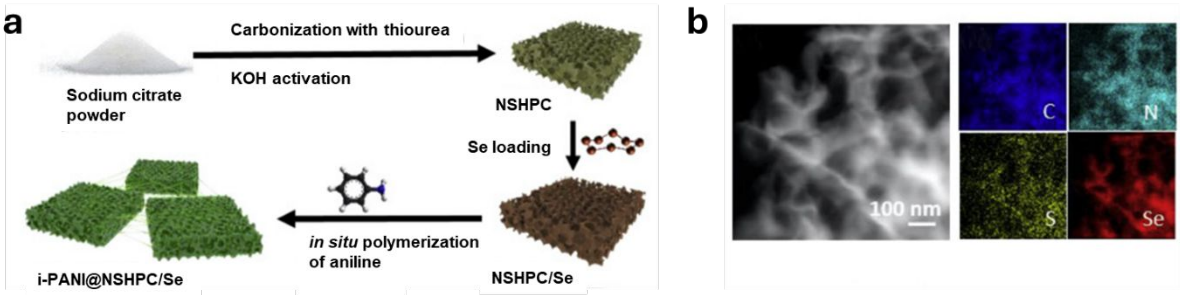

Figure S2| **a** Preparation of aniline-based polymer and Se-composite. **b** HR-TEM and EDS maps of C, N, S, Se.[4] Reproduced with permission from ref.4. Copyright 2019, Elsevier

(4) Zhang, F.; Xiong, P.; Guo, X.; Zhang, J.; Yang, W.; Wu, W.; Liu, H.; Wang, G. A Nitrogen, Sulphur Dual-Doped Hierarchical Porous Carbon with Interconnected Conductive Polyaniline Coating for High-Performance Sodium-Selenium Batteries. *Energy Storage Mater.* **2019**, *19*, 251–260. https://doi.org/10.1016/j.ensm.2019.03.019.

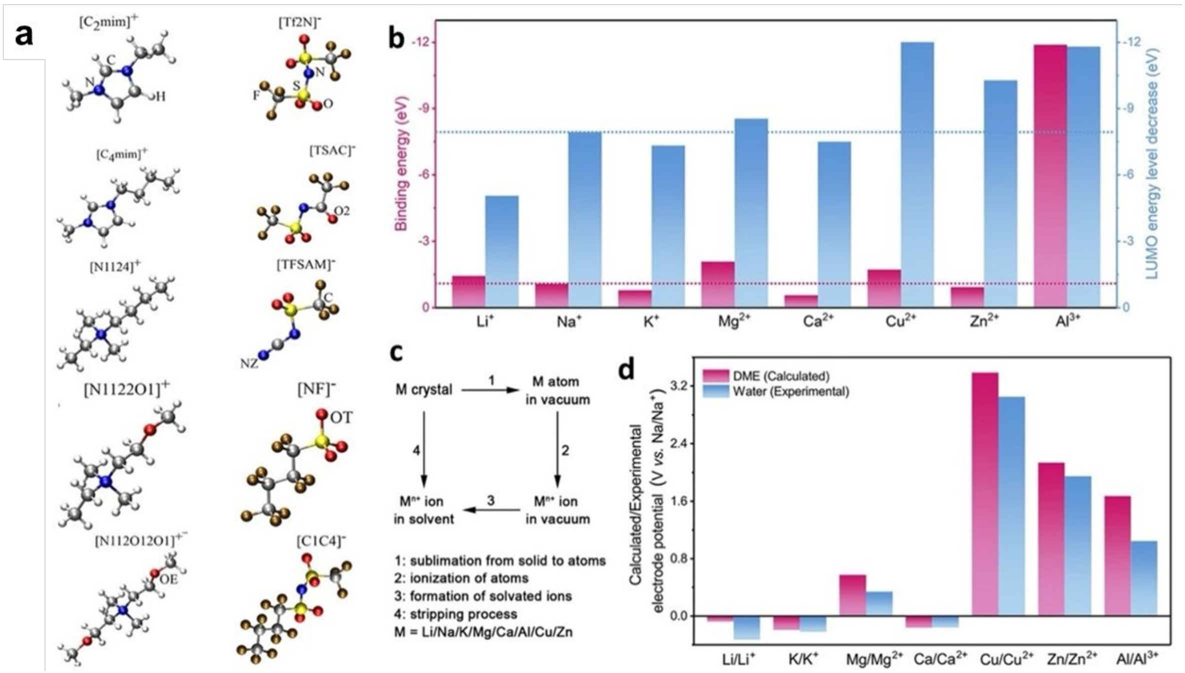

Figure S3| **a** Atomic structures of ILs obtained by DFT calculations. The C, O, H, S, F, and N atoms are indicated by silver, red, white, yellow, gold, and blue spheres, respectively.[5] (Reproduced with permission from ref. 5. Copyright 2021, American Chemical Society) **b** Comparison of the LUMO energy level decreases and binding energy of ion-DME complexes. **c** The thermodynamic cycle of calculating electrode potential. **d** Calculated and experimental electrode potentials of the considered metal/cation pairs.[6] Reproduced with permission from ref. 6. Copyright 2020, Elsevier.

(5) Lourenço, T. C.; Dias, L. G.; Da Silva, J. L. F. Theoretical Investigation of the Na^+ Transport Mechanism and the Performance of Ionic Liquid-Based Electrolytes in Sodium-Ion Batteries. *ACS Appl. Energy Mater.* **2021**, *4* (5), 4444–4458, https://doi.org/10.1021/acsaem.1c00059.

(6) Chen, X.; Shen, X.; Hou, T. Z.; Zhang, R.; Peng, H. J.; Zhang, Q. Ion-Solvent Chemistry-Inspired Cation-Additive Strategy to Stabilize Electrolytes for Sodium-Metal Batteries. *Chem.* **2020**, *6* (9), 2242–2256, https://doi.org/10.1016/j.chempr.2020.06.036.

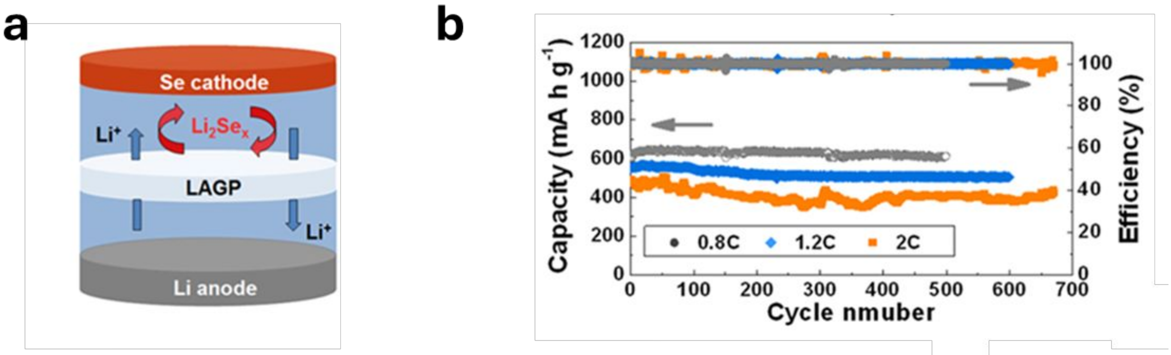

Figure S4| **a** Schematic illustration of the Li-Se battery with hybrid electrolytes.[7] **b** Capacity and coulombic efficiency of Li-Se batteries with hybrid electrolytes (with $LiNO_3$).[7] Reproduced with permission from ref. 7. Copyright 2017, Elsevier.

(7) Zhou, Y.; Li, Z.; Lu, Y. C. A Stable Lithium–Selenium Interface via Solid/Liquid Hybrid Electrolytes: Blocking Polyselenides and Suppressing Lithium Dendrite. *Nano Energy* **2017**, *39*, 554–561. https://doi.org/10.1016/j.nanoen.2017.07.038.

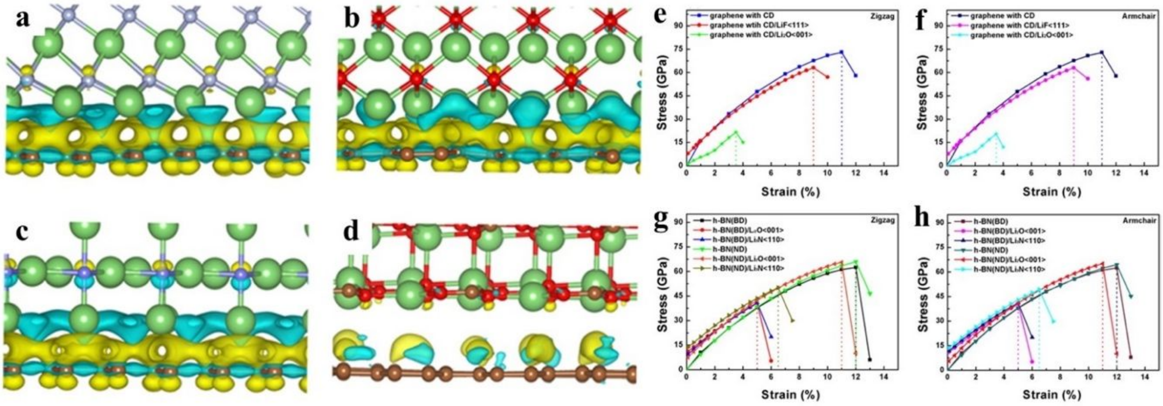

Figure S5| The differential charge density of **a** graphene/LiF<111>, **b** graphene/Li_2O<111>, **c** graphene/Li_3N<001>, **d** graphene/Li_2CO_3<001>. Tensile stress as a function of bi-axial strain for four kinds of BL-SEIs: **e** and **f** show evolution profiles for graphene/LiF<111>and graphene/Li_2O<001>with CD along zigzag and armchair directions respectively, **g** and **h** exhibit evolution profiles for h-BN/Li_2O<001>and h-BN/Li_3N<110>with both BD and ND along zigzag and armchair directions respectively.[8] Reproduced with permission from ref. 8. Copyright 2019, Elsevier.

(8) Zhu, J.; Li, P.; Chen, X.; Legut, D.; Fan, Y.; Zhang, R.; Lu, Y.; Cheng, X.; Zhang, Q. Rational Design of Graphitic-Inorganic Bi-Layer Artificial SEI for Stable Lithium Metal Anode. *Energy Storage Mater.* **2019**, *16*, 426–433, https://doi.org/10.1016/j.ensm.2018.06.023. Figure 2a-d, Figure 5 a-d

Table S1: Comparison of advantages and disadvantages of different electrolyte systems: key points for carbonate, ether, solid-state and hybrid liquid electrolytes.

**Electrolyte system**	**Advantages**	**Disadvantages**
Carbonate-Based	-High thermal stability<br>-Widely used and well-studied<br>-Good compatibility with Li and Na anodes	-Lower ionic conductivity compared to ethers<br>-Slow reaction kinetics with Se<br>-Challenges in forming a stable SEI layer on metal anodes
Ether-Based	-Higher ionic conductivity than carbonates<br>-Enhanced compatibility with Se due to solubility of intermediates<br>- Better formation of stable SEI layers, preventing dendrite growth	-Susceptible to shuttle effects due to polyselenide dissolution<br>-Lower electrochemical stability<br>-Safety concerns due to flammability
Solid-State	-Excellent chemical and thermal stability<br>-No dendrite formation, enhancing safety<br>-Prevents polyselenide shuttle effects	-High interfacial resistance and mechanical issues<br>-Poor ionic conductivity at room temperature<br>-Expensive and complex manufacturing
Hybrid (liquid+solid)	-Combines the safety of solids with the high conductivity of liquids<br>-Better flexibility in electrolyte design<br>-Potential for stable SEI layer with high ionic conductivity	-Interface stability between liquid and solid components can be challenging<br>-More complex electrolyte management and synthesis<br>-Limited practical data for large-scale applications

Table S2: Comparison of Li-Se, Na-Se and K-Se based systems in terms of theoretical capacity, energy density, cycle-life, polyselenide solubility, diffusion coefficient and redox kinetics.[9–15]

Parameter	Li-Se	Na-Se	K-Se
Theoretical capacity	~675 mAh/g	~678 mAh/g	~750 mAh/g
Energy density	High (~2530 Wh/kg)	Moderate (~2000 Wh/kg)	Moderate (~1800 Wh/kg)
Cycle life	Moderate to High (300+ cycles)	Moderate (200-300 cycles)	Moderate (100-200 cycles)
Polyselenide solubility	Moderate, soluble in ether-based electrolytes	High, leading to a pronounced shuttle effect	High, leading to fast dissolution and shuttle effect
Diffusion coefficient	Moderate	High	Very High
Redox kinetics	Moderate (relatively higher energy barriers)	Fast (lower energy barriers facilitate redox reactions)	Faster (lowest energy barriers among the three systems)
Advantages	High energy density, stable SEI with optimized electrolytes	Faster ion diffusion, cost-effective, abundant sodium	Excellent rate capability, cost-effective, abundant potassium
Challenges	Shuttle effect, dendrite formation on Li anodes	Shuttle effect, rapid polyselenide dissolution	Short cycle life, high polyselenide dissolution, severe shuttle effect

(9) Luo, C.; Xu, Y.; Zhu, Y.; Liu, Y.; Zheng, S.; Liu, Y.; Langrock, A.; Wang, C. Selenium@Mesoporous Carbon Composite with Superior Lithium and Sodium Storage Capacity. *ACS Nano* **2013**, *7* (9), 8003–8010, https://doi.org/10.1021/nn403108w.

(10) Sun, J.; Du, Z.; Liu, Y.; Ai, W.; Wang, K.; Wang, T.; Du, H.; Liu, L.; Huang, W. State-Of-The-Art and Future Challenges in High Energy Lithium–Selenium Batteries. *Adv. Mater.* **2021**, *33*, 2003845, https://doi.org/10.1002/adma.202003845.

(11) Xu, R.; Cheng, X. B.; Yan, C.; Zhang, X. Q.; Xiao, Y.; Zhao, C. Z.; Huang, J. Q.; Zhang, Q. Artificial Interphases for Highly Stable Lithium Metal Anode. *Matter* **2019**, *1*, 317–344, https://doi.org/10.1016/j.matt.2019.05.016.

(12) Manthiram, A.; Fu, Y.; Chung, S. H.; Zu, C.; Su, Y. S. Rechargeable Lithium-Sulfur Batteries. *Chem. R.* **2014**, *114*, 11751–11787, https://doi.org/10.1021/cr500062v.

(13) Ye, H.; Yin, Y. X.; Zhang, S. F.; Guo, Y. G. Advanced Se-C Nanocomposites: A Bifunctional Electrode Material for Both Li-Se and Li-Ion Batteries. *J. Mater. Chem. A* **2014**, *2 (33)*, 13293–13298, https://doi.org/10.1039/c4ta02017k.

(14) Gates, B.; Yin, Y.; Xia, Y. A Solution-Phase Approach to the Synthesis of Uniform Nanowires of Crystalline Selenium with Lateral Dimensions in the Range of 10-30 nm. *J. Am. Chem. Soc.* **2000**, *122*, 12582–12583, https://doi.org/10.1021/ja002608d.

(15) Huang, H.; Xu, R.; Feng, Y.; Zeng, S.; Jiang, Y.; Wang, H.; Luo, W.; Yu, Y. Sodium/Potassium-Ion Batteries: Boosting the Rate Capability and Cycle Life by Combining Morphology, Defect and Structure Engineering. *Adv. Mater.* **2020**, *32* (8), 1904320, https://doi.org/10.1002/adma.201904320.
